# Elevated CCL2 causes Leydig cell malfunction in metabolic syndrome

**DOI:** 10.1172/jci.insight.134882

**Published:** 2020-11-05

**Authors:** Qingkui Jiang, Constanze C. Maresch, Sebastian Friedrich Petry, Agnieszka Paradowska-Dogan, Sudhanshu Bhushan, Yongsheng Chang, Christine Wrenzycki, Hans-Christian Schuppe, Petr Houska, Michaela F. Hartmann, Stefan A. Wudy, Lanbo Shi, Thomas Linn

**Affiliations:** 1 Clinical Research Unit, Centre of Internal Medicine, Justus-Liebig-University (JLU), Giessen, Germany.; 2 Department of Gynecological Endocrinology and Reproductive Medicine, University Clinic Bonn, Bonn, Germany.; 3 Institute of Anatomy and Cell Biology, Department of Reproductive Biology, JLU, Giessen, Germany.; 4 Tianjin Key Laboratory of Cellular and Molecular Immunology, Key Laboratory of Immune Microenvironment and Disease (Ministry of Education), Department of Physiology and Pathophysiology, Tianjin Medical University, Tianjin, China.; 5 Department of Molecular Reproductive Medicine, Clinic for Veterinary Obstetrics, Gynecology and Andrology, and; 6 Department of Urology, Pediatric Urology and Andrology, JLU, Giessen, Germany.; 7 ANOVA, Karolinska University Hospital and Karolinska Institutet, Stockholm, Sweden.; 8 Steroid Research and Mass Spectrometry Unit, Division of Pediatric Endocrinology and Diabetology, Center of Child and Adolescent Medicine, JLU, Giessen, Germany.; 9 Public Health Research Institute, New Jersey Medical School, Rutgers Biomedical and Health Sciences, Rutgers, The State University of New Jersey, Newark, New Jersey, USA.

**Keywords:** Endocrinology, Reproductive Biology, Chemokines, Fertility, Urology

## Abstract

Metabolic syndrome (MetS), which is associated with chronic inflammation, predisposes males to hypogonadism and subfertility. The underlying mechanism of these pathologies remains poorly understood. Homozygous leptin-resistant obese *db/db* mice are characterized by small testes, low testicular testosterone, and a reduced number of Leydig cells. Here we report that IL-1β, CCL2 (also known as MCP-1), and corticosterone concentrations were increased in the testes of *db/db* mice relative to those in WT controls. Cultured murine and human Leydig cells responded to cytokine stress with increased CCL2 release and apoptotic signals. Chemical inhibition of CCL2 rescued Leydig cell function in vitro and in *db/db* mice. Consistently, we found that *Ccl2*-deficient mice fed with a high-energy diet were protected from testicular dysfunction compared with similarly fed WT mice. Finally, a cohort of infertile men with a history of MetS showed that reduction of CCL2 plasma levels could be achieved by weight loss and was clearly associated with recovery from hypogonadism. Taken together, we conclude that CCL2-mediated chronic inflammation is, to a large extent, responsible for the subfertility in MetS by causing damage to Leydig cells.

## Abstract 2

MCP-1/CCL2 upregulation associates with metabolic syndrome–induced male subfertility in both mice and men.

## Introduction

Infertility affects 10% of couples worldwide, and male factors contribute to more than 40% of the infertility cases (1, 2). Metabolic syndrome (MetS) is a cluster of abnormalities including type 2 diabetes, obesity, dyslipidemia, and hypertension that has been proposed as a potential cause of male infertility (3–6). MetS has been associated with a higher incidence of male infertility compared with healthy men, as indicated by decreased serum testosterone/estrogen ratio and poor semen quality. However, the mechanisms underlying the MetS-induced male reproductive disorders remain unclear (7–12).

The testis is the male gonad with functions of spermatogenesis and androgen production. Inflammation within the testis has been postulated as an important etiological factor of male infertility (13–16). Disrupted spermatogenesis has been reported when orchitis is induced by lipopolysaccharide (LPS) injection (17). Although not as highly elevated as compared with severe infection, increased blood levels of cytokines are characteristics of MetS (18, 19). In adipose and liver tissue, enhanced expression of tumor necrosis factor–α (TNF-α), interleukin-1β (IL-1β), interleukin-6 (IL-6), and C-C motif chemokine ligand 2 (CCL2), also known as monocyte chemoattractant protein-1 (MCP-1), was reported (20–23). These inflammatory factors are causative and predictive for many organ-specific diseases associated with MetS (24), yet the mechanism linking chronic inflammation to testicular impairment in the setting of MetS has not been addressed.

Testicular interstitium contains several cell types, including Leydig cells, macrophages, and endothelial cells, capable of producing proinflammatory cytokines such as IL-1β, IL-6, and TNF-α to modulate testis functions (25–29). CCL2 is a chemokine expressed constitutively in testis by Leydig cells and peritubular cells (30, 31). In obese subjects, increased abundance of CCL2 was observed in both white adipose tissue and plasma (32, 33). Mouse models have also demonstrated that the expression of CCL2 increases with body weight increase and contributes to insulin resistance (34, 35). By contrast, CCL2 deficiency abrogates inflammation and steatosis secondary to high-fat diet (36). Elevated CCL2 level in the testis of experimental autoimmune epididymo-orchitis indicates that CCL2 might regulate testicular functions in inflammation (37); however, its potential roles have not been explored.

The C57BLKS-*Lepr^db^* mouse (*db/db* mouse) is homozygous for a mutation in its leptin receptor (*Lepr*) gene and is a well-established animal model for type 2 diabetes and MetS. Male *db/db* mice fail to reproduce, but testicular factors associated with the full MetS phenotype, including type 2 diabetes, have not been investigated to our knowledge. A neuron-specific transgenic expression of the *Lepr* transgene rescues the MetS phenotype and reproductive capacity of male *db/db* mice, implying that the MetS phenotype, rather than testicular deficiency of *Lepr*, leads to infertility in male *db/db* mice (38).

Based on these considerations, we hypothesized that proinflammatory cytokines in the testis of *db/db* mice would play an important role in reflecting conditions of human MetS. We examined testes in diabetic *db/db* mice with fully manifested MetS and identified Leydig cells as the foremost target of obesity-induced inflammation. We then established various other mouse and cell culture models to dissect male factor subfertility in MetS and the role of inflammation in it. In addition, a clinical trial in infertile men was performed to further test our hypothesis and explore potential treatment strategies.

## Results

### MetS and male infertility.

**Figure 1:**
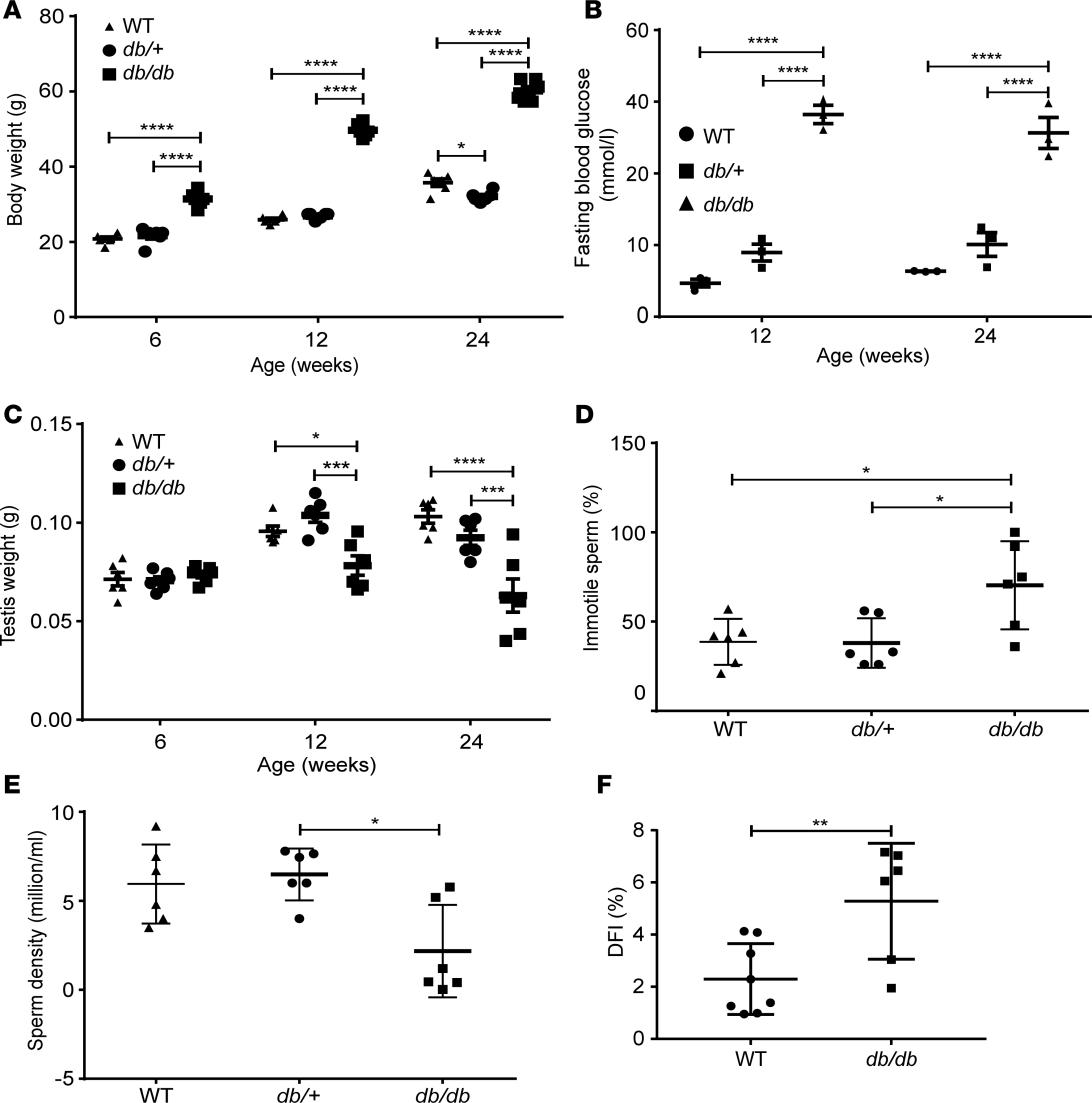
Characteristics of phenotype and reproductivity of *db/db* mice. (**A**) Body weight of WT, *db/+*, and *db/db* mice (*n* = 6) at 6, 12, and 24 weeks of age. (**B**) Blood glucose levels in WT, *db/+*, and *db/db* mice at 12 and 24 weeks of age (*n* = 6). (**C**) Testis weight of WT, *db/+*, and *db/db* mice at 6, 12, and 24 weeks of age; *n* = 3 in each group. (**D** and **E**) Sperm motility (**D**) and density (**E**) in 12- to 24-week-old mice; *n* = 6 in each group. (**F**) DNA fragmentation index (DFI) of epididymal sperm from age-matched 12- to 24-week-old *db/db* (*n* = 6) and WT (*n* = 7) mice. Data represent 1 of 3 independent experiments and are shown as means ± standard error of the mean (SEM). Student’s 2-tailed *t* test was used to compare means between 2 groups, and 1-way ANOVA was used to compare means between 3 groups followed by Tukey’s post hoc comparisons. *P < 0.05, **P < 0.01, ***P < 0.001, ****P < 0.0001.

*db/db* mice showed significantly higher body weight at 6 weeks of age compared with that of WT and BKS(D)-*Lepr*^db+/–^/JOrlRj (*db/+*) (*db/+*) mice (Figure 1A and Supplemental Figure 1A; supplemental material available online with this article; https://doi.org/10.1172/jci.insight.134882DS1). Fasting blood glucose levels of *db/db* mice were markedly higher than those of WT and *db/+* mice at 12 and 24 weeks (Figure 1B). To investigate the reproductive function in these mice, testis weights were assessed at different ages, and sperm parameters were measured at 24 weeks. The testis of *db/db* mice had comparable weight (Figure 1C) and histology (Supplemental Figure 1B) as *db/+* and WT mice at 6 weeks. However, *db/db* mice showed significantly lower testis weight than that of WT and *db/+* mice at 12 and 24 weeks (Figure 1C), indicating testicular disturbance developed with progression of full MetS. Consistently, *db/db* mice had reduced seminiferous tubule diameter (Supplemental Figure 1C) and seminiferous epithelium thickness (Supplemental Figure 1D), disturbed spermatogenesis (Supplemental Figure 1E), and increased germ cell apoptosis (Supplemental Figure 2). Moreover, epididymal sperm from *db/db* mice showed poor motility, 70% ± 10% *db/db* sperm vs. 39% ± 5% WT sperm, and 38% ± 6% *db/+* sperm were immobile (*P* < 0.05, Figure 1D). The sperm density declined from 6.0 ± 0.9 × 10^6^/mL in WT mice and 6.5 ± 0.6 × 10^6^/mL in *db/+* to 2.2 ± 1.1 × 10^6^/mL in *db/db* mice (*P* < 0.01, Figure 1E). Furthermore, the DNA fragmentation index (DFI) indicated impaired DNA integrity in sperm from *db/db* mice (*P* < 0.01, Figure 1F), which could be responsible for the complete failure of reproduction of *db/db* males as compared with WT males in controlled breeding experiments (0 vs. 5.75 ± 0.75 litters) (Table 1). Concordantly, developmental rates of oocytes fertilized in vitro with sperm from *db/db* mice up to the morula/blastocyst stage were significantly lower than those of oocytes fertilized with WT sperm (Table 2). Because male *db/+* mice have been characterized by normal fertility in various studies (38–42), we focused on comparing *db/db* with WT mice in the following experiments unless stated otherwise.

### Loss of Leydig cells.

**Figure 2:**
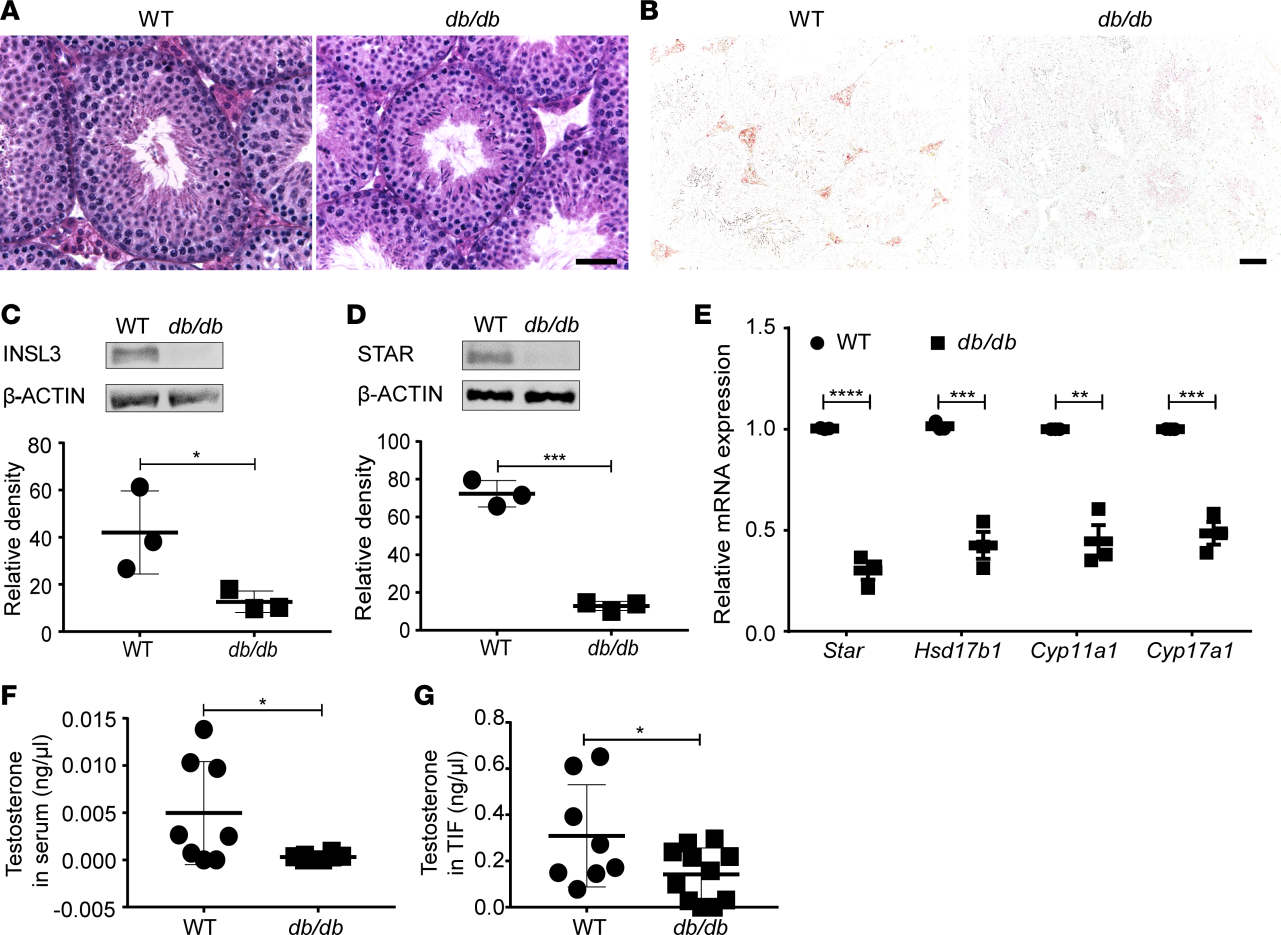
*db/db* mice showed impaired expression of specific Leydig cell products. (**A** and **B**) H&E (**A**) and insulin-like 3 (INSL3) (**B**) staining on representative testicular sections; scale bar: 20 μm. (**C** and **D**) Protein expression of INSL3 (**C**) and STAR (**D**), respectively, as analyzed by Western blot. (**E**) mRNA levels of steroidogenic genes in WT and *db/db* mice. (**F** and **G**) Mean testosterone levels in serum (**F**) and testicular interstitial fluid (TIF) (**G**). Data given in **C**–**G** were from *n* = 3–8 mice, 12–24 weeks old, in each group. Data are shown as means ± SEM. Student’s 2-tailed *t* test was used to compare means between 2 groups. *P < 0.05, **P < 0.01, ***P < 0.001, ****P < 0.0001.

The most conspicuous phenotype in testis sections of *db/db* mice was a decrease of Leydig cell numbers (Figure 2A). Immunostaining of INSL3, a peptide uniquely produced by Leydig cells, revealed a total loss of its expression in the testis of *db/db* mice at 12–24 weeks (Figure 2B), which was further confirmed by quantitative Western blot analysis (Figure 2C). Significant reduction of steroidogenic acute regulatory protein (STAR), a rate-limiting enzyme in the production of androgen, was observed at both the protein (Figure 2D) and mRNA levels (Figure 2E) along with the reduction of mRNA levels of other steroidogenic genes (*Hsd17b1*, *Cyp11a1*, and *Cyp17a1*) in the testis of *db/db* mice (Figure 2E). Consequently, testosterone levels in both serum and testicular interstitial fluid (TIF) of *db/db* mice were markedly reduced compared with those of WT mice (Figure 2, F and G). These results suggest that Leydig cells, known as the primary source of androgens, were reduced in number with potentially impaired function in the testis of *db/db* mice.

### Expression of fertility genes and testicular cytokine activity.

**Figure 3:**
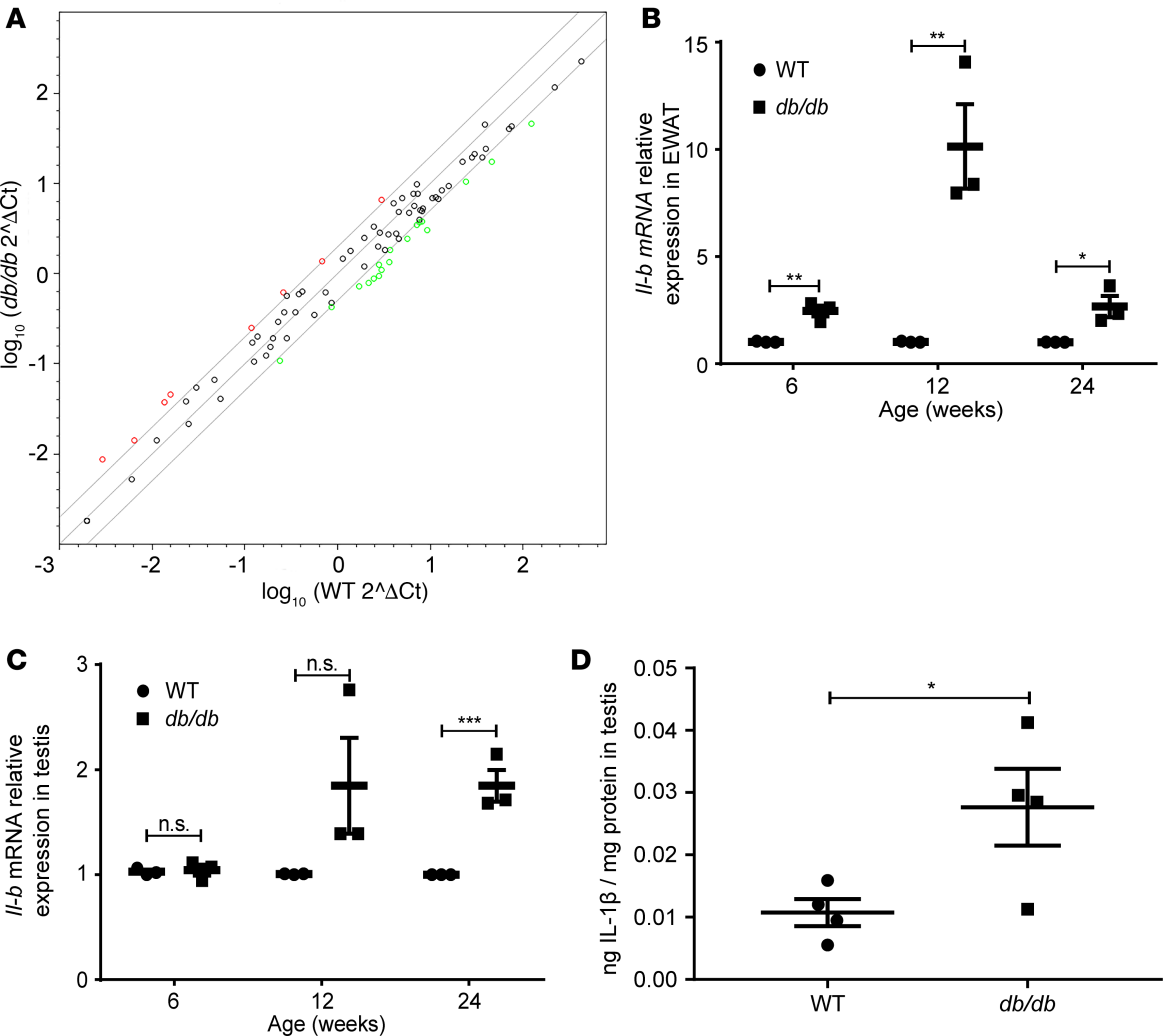
The expression of proinflammatory factors in testes of WT and *db/db* mice. (**A**) Log transformation plot of the relative gene expression levels in testes of *db/db* compared with in testes of WT mice. Colored dots indicate 2-fold or greater changes in mRNA levels (red, reregulation; green, downregulation). Samples were pooled from 4 mice in each group. (**B**) The *Ilb* mRNA levels in epididymal white adipose tissue (EWAT). *N* = 3 in each group. (**C**) The *Ilb* mRNA levels in testes. *N* = 3 in each group. (**D**) IL-1β protein in testes. *N* = 4 in each group. Data represent 1 of 3 independent experiments and are shown as means ± SEM. Student’s 2-tailed *t* test was used to compare means between 2 groups. *P < 0.05, **P < 0.01, ***P < 0.001.

We next examined whether inflammatory cytokines were involved in infertility caused by MetS. Quantitative real-time PCR (RT-qPCR) analysis of mRNA isolated from testicular tissue showed downregulation of genes related to spermatogenesis (*Acsbg2*, *Adcy10*, *Catsper1*, *Catsper4*, and *Crisp2*) and fertilization (*Akap4*, *Trim36*, and *Plcz1*) and upregulation of genes associated with oxidative stress (*Cdo1*, *Fos*, and *Uchl1*) in the testis of *db/db* mice (Figure 3A and Table 3). Notably, elevated *Il16* and *Nfκbia* mRNA indicated the activation of inflammatory pathways. Analysis of the expression of other proinflammatory markers revealed *Ilb* as the only gene measured with increased expression in both adipose tissue (Figure 3B) and testes (Figure 3C, Supplemental Figure 3) of *db/db* mice. Elevation of testicular IL-1β protein level in *db/db* mice, as compared with WT mice, was confirmed by ELISA using whole testis lysates (Figure 3D). These results imply that IL-1β–mediated inflammation might play a causative role in the pathogenesis of subfertility in *db/db* mice.

### Upregulation of CCL2.

**Figure 4:**
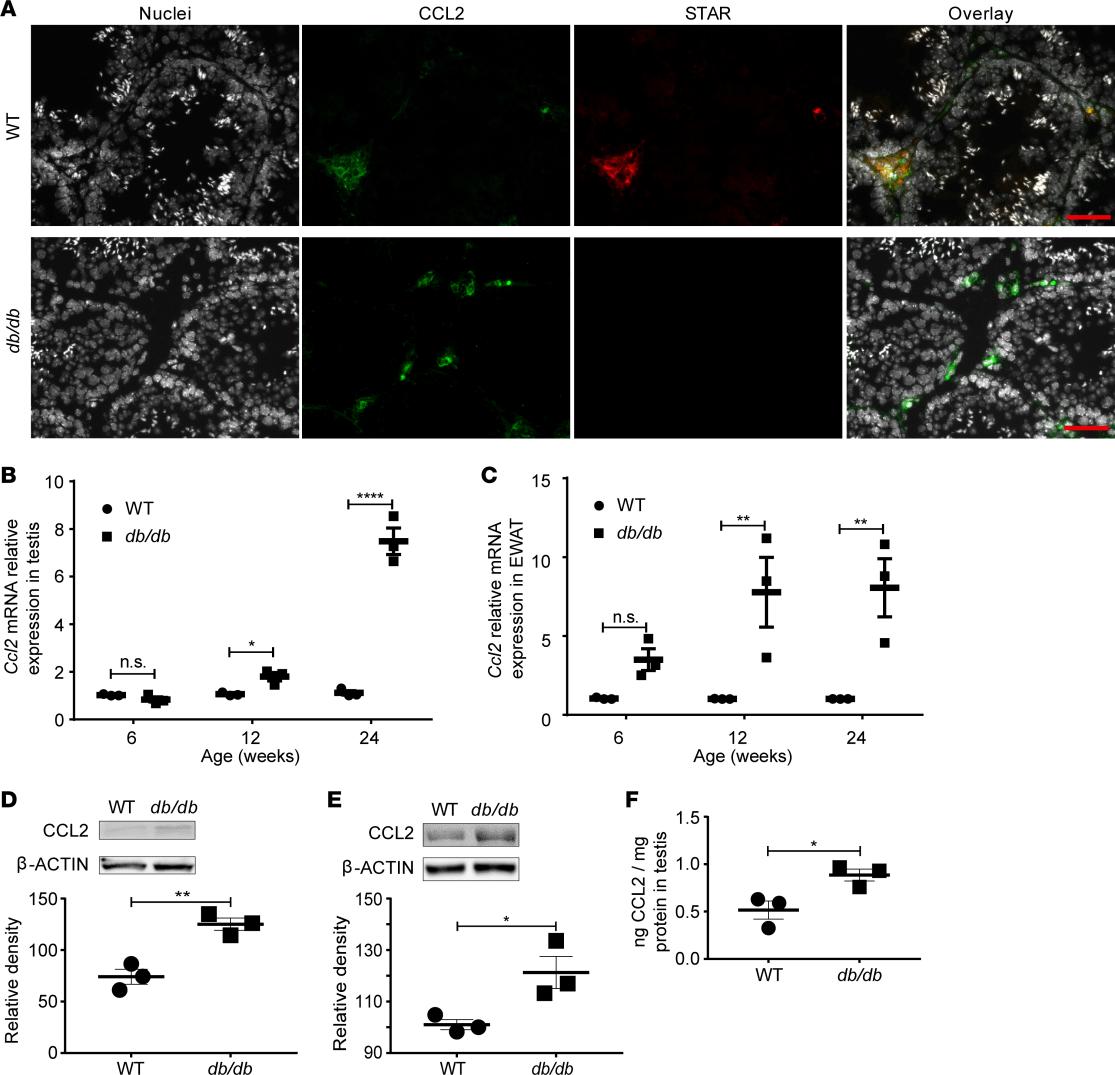
CCL2 expression was increased in *db/db* mice. (**A**) Representative sections show colocalization of CCL2 in STAR-positive cells of WT controls. Scale bar: 20 μm. CCL2 is present in STAR-negative cells close to the basal membrane of the tubuli. (**B**) The *Ccl2* mRNA levels in testes. *N* = 3 in each group. (**C**) The *Ccl2* mRNA levels in EWAT. *N* = 3 in each group. (**D**) CCL2 protein in testes as determined by Western blot and followed by quantification. *N* = 3 in each group. (**E**) CCL2 protein expression in EWAT as determined by Western blot and followed by quantification. *N* = 3 in each group. (**F**) CCL2 protein concentration in testes as determined by ELISA. *N* = 3 in each group. Panels **A**, **D**, and **E** show representative images of 1 analysis per mouse. Data represent 1 of 3 independent experiments and are shown as means ± SEM. Student’s 2-tailed *t* test was used to compare means between 2 groups. *P < 0.05, **P < 0.01, ****P < 0.0001.

Because CCL2 was also suggested to be involved in MetS, expression of this proinflammatory chemokine was investigated. First, we examined the distribution of CCL2 protein in the testis. Immunofluorescence staining of WT testis revealed that Leydig cells had the capacity to produce substantial amounts of CCL2, as demonstrated by double staining of STAR and CCL2 (Figure 4A). Because no STAR-positive cells were observed, but CCL2 protein was still detected in the testis of *db/db* mice at 12–24 weeks, it is evident that CCL2 was expressed by testicular cells other than Leydig cells, presumably macrophages and/or peritubular cells. CCL2 might thus be a paracrine factor that contributes to Leydig cell loss in *db/db* mice. A significant increase of CCL2 was confirmed at mRNA and protein levels in both testes and adipose tissue (Figure 4, B–F) of *db/db* mice at 12–24 weeks of age.

### Macrophage profile changes associated with MetS.

**Figure 5:**
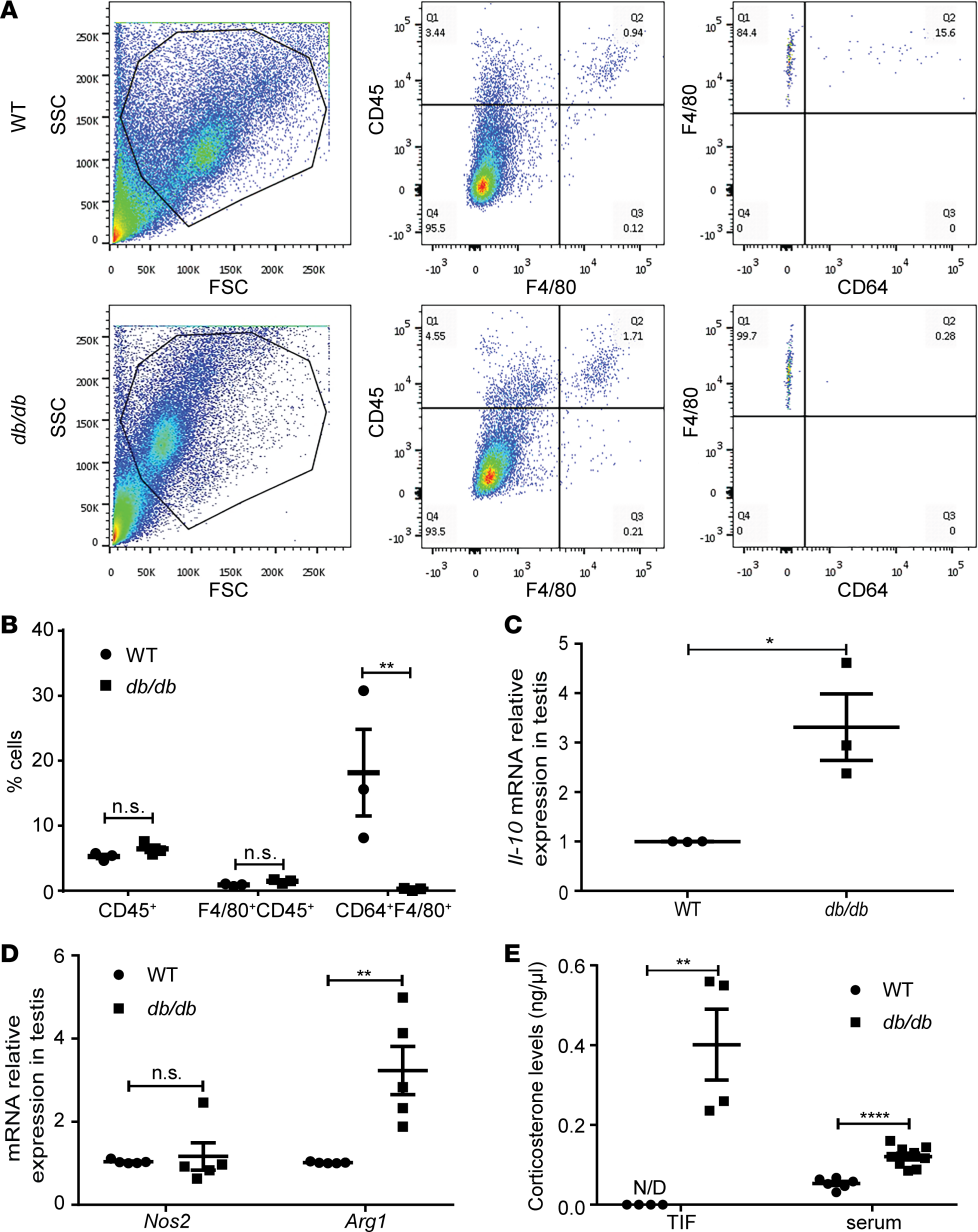
Testicular immune cells and corticosterone levels in *db/db* mice. (**A**) Total testicular cells were isolated as described in Methods and examined by FACS. Representative flow cytometry gating schemes for testicular cells from WT and *db/db* mice are demonstrated. The gating strategy is shown for CD45^+^F4/80^+^ and F4/80^+^CD64^+^ cells. (**B**) Frequencies of testicular F4/80^+^ within CD45^+^ cells and CD64^+^ within F4/80^+^CD45^+^ cells from WT and *db/db* mice. (**C**) The *Il-10* mRNA expression in testes. (**D**) The *Nos2* and *Arg1* mRNA expression in testes. (**E**) Corticosterone levels in TIF and serum. *N*= 3–8 mice, 12 to 24 weeks old, in each group. Data are shown as means ± SEM. Student’s 2-tailed *t* test was used to compare means between 2 groups. *P < 0.05, **P < 0.01, ****P < 0.0001.

Because Leydig cells are positionally and functionally associated with testicular macrophages, we reasoned that properties of resident macrophages might be a factor influencing Leydig cells’ viability. We therefore analyzed macrophage populations in the testis by flow cytometry (Figure 5, A and B). Although the percentage of F4/80^+^CD45^+^ was not significantly different between testis of *db/db* and WT mice, the proportion of proinflammatory CD64^+^F4/80^+^ macrophages was reduced in *db/db* mice (Figure 5, A and B). F4/80 immunohistochemical staining showed a clear reduction of macrophages in the testis of *db/db* mice (Supplemental Figure 4), indicating that the absolute F4/80^+^ macrophages were reduced proportionally to the total cell number in testis of *db/db* mice, which resulted in the comparable percentage of F4/80^+^CD45^+^ in the testis of *db/db* and WT mice. Increased *Il-10* mRNA level in the testis of *db/db* mice suggested a shift of macrophage population to the antiinflammatory M2 phenotype (Figure 5C), which was supported by the upregulated arginase 1 (*Arg1*) mRNA level and comparable nitric oxide synthase 2 (*Nos2*) levels in the testis of *db/db* mice as compared with those in the testis of WT mice (Figure 5D). Interestingly, while corticosterone was undetectable in the TIF of WT mice, its level in *db/db* mice was significantly increased in both serum and TIF (Figure 5E).

### CCL2 expression is induced by IL-1β in vitro.

**Figure 6:**
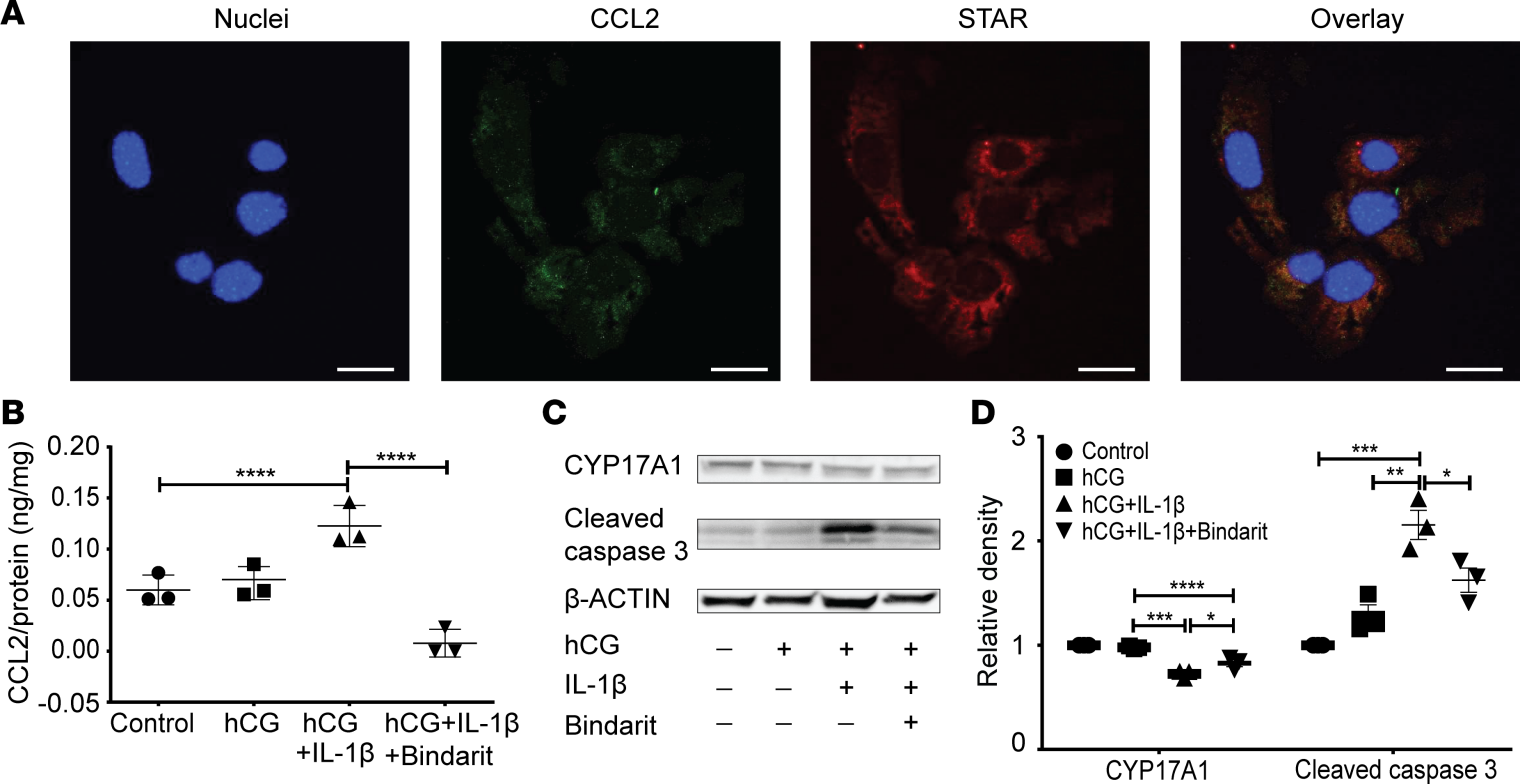
CCL2 regulated function and apoptosis of MLTC-1 cells. (**A**) Representative pictures showing MLTC-1 cells express CCL2 under basal cultivating conditions. (**B**) Amount of CCL2 in the culture medium. (**C** and **D**) Western blot analysis of CYP17A1 and cleaved caspase-3 in MLTC-1 cells after 48 hours’ treatment with human chorionic gonadotropin (hCG) in the absence or presence of IL-1β (1 ng/mL), with or without the CCL2 inhibitor Bindarit (100 μM). *N* = 3 in each group. Data represent one of 3 independent experiments and are shown as means ± SEM. One-way ANOVA was used to compare means between groups followed by Tukey’s post hoc comparisons. *P < 0.05, **P < 0.01, ****P < 0.0001.

**Figure 7:**
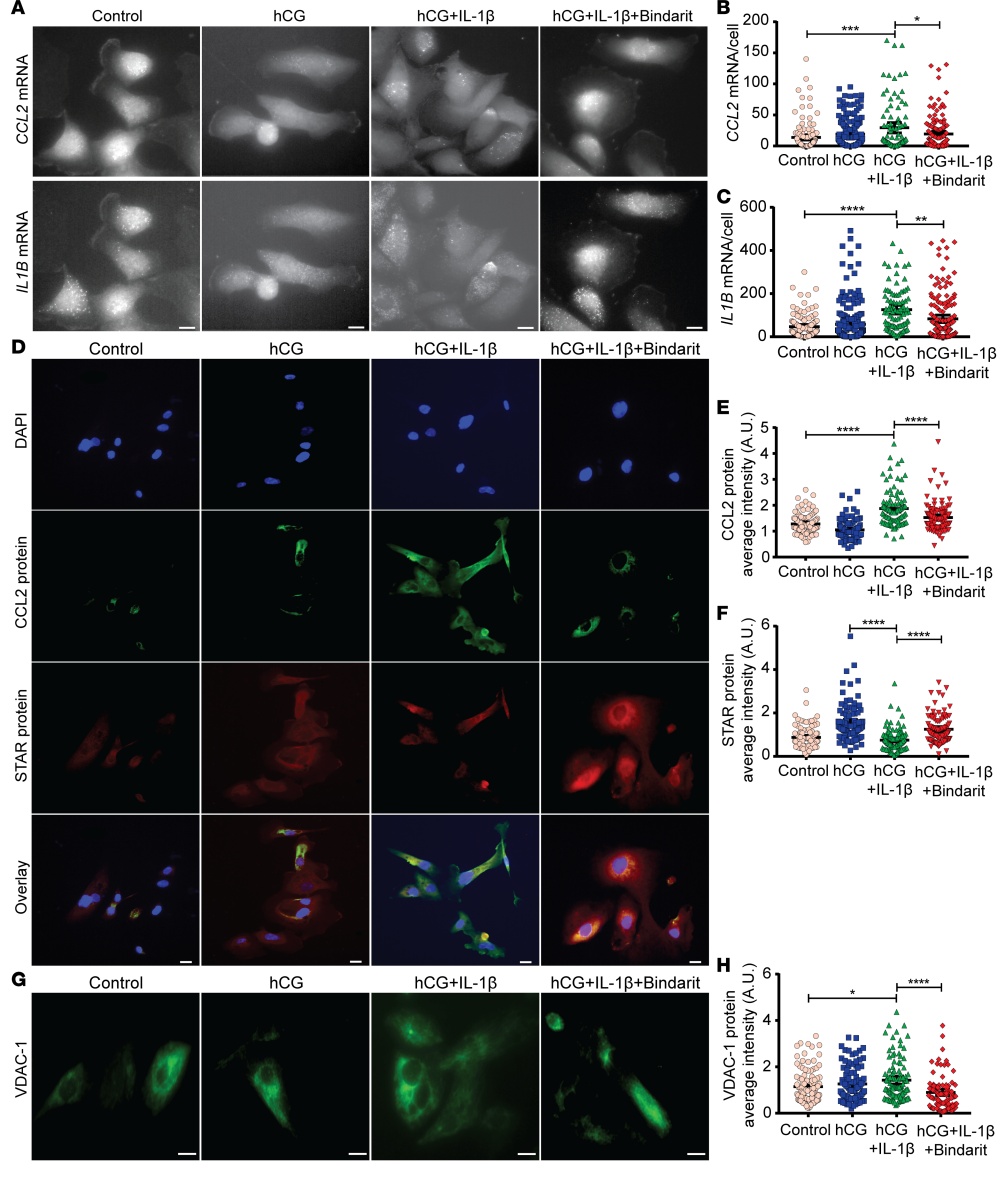
CCL2 regulated function and apoptosis of primary human Leydig cells. (**A**–**C**) Representative pictures showing *CCL2* and *ILB* mRNA expressions in HLC after 48 hours’ treatment with hCG in the absence or presence of IL-1β (1 ng/mL), with or without Bindarit (100 μM) (**A**), followed by quantification: number of *CCL2* (**B**) and *ILB* (**C**) mRNA molecules per HLC. (**D**–**F**) Representative pictures showing CCL2 and STAR protein expressions in HLC after 48 hours’ treatment with hCG in the absence or presence of IL-1β (1 ng/mL), with or without Bindarit (100 μM) (**D**), followed by quantification: average fluorescence intensity of CCL2 (**E**) and STAR (**F**) per HLC. (**G** and **H**) Representative pictures showing voltage-dependent anion-selective channel 1 (VDAC-1) protein expression in HLC after 48 hours’ treatment with hCG in the absence or presence of IL-1β (1 ng/mL), with or without Bindarit (100 μM) (**G**), followed by quantification, average fluorescence intensity of VDAC-1 per HLC (**H**). Data represent at least 3 independent experimental runs. Scale bar: 10 μm. *N* = ~100–180 cells in each group. Data represent 1 of 3 independent experiments and are shown as means ± SEM. One-way ANOVA was used to compare means between groups followed by Tukey’s post hoc comparisons. *P < 0.05, **P < 0.01, ***P < 0.001, ****P < 0.0001.

Reduced inflammatory macrophages in the testis of *db/db* mice indicated that different sources were responsible for the increased CCL2 expression in the testis. One of the candidates would be Leydig cells because of their constitutive expression of CCL2 (30, 31), which might be increased in the presence of proinflammatory cytokines. To test this hypothesis, we mimicked the testicular microenvironment by treating the murine Leydig cell line MLTC-1 (Figure 6) and primary human Leydig cells (HLC) (Figure 7) with hCG and/or IL-1β. First, we showed that CCL2 is constitutively expressed in MLTC-1 cells (Figure 6A). Addition of IL-1β (1 ng/mL) significantly induced CCL2 protein levels along with decreased expression of a steroidogenic enzyme, CYP17A1, in MLTC-1 cells (Figure 6, B–D). These changes were accompanied by induction of apoptosis, as measured by cleaved caspase-3 levels in MLTC-1 (Figure 6, C and D). Similarly, IL-1β induced proinflammatory response in HLC, as evidenced by increased CCL2 (at the mRNA and protein levels) and *Ilb* (on the mRNA level) expression, and inhibited STAR protein expression (Figure 7, A–F). The elevated VDAC-1 level in HLC after IL-1β treatment indicated onset of apoptosis (Figure 7, G and H) (43). Of note, activities of steroidogenic enzymes, IL-1β induction, and apoptotic events were partially rescued by administrating the CCL2 inhibitor Bindarit (Figure 7, A–H). These findings demonstrate that CCL2 induced by IL-1β was a proapoptotic factor for Leydig cells.

### CCL2 inhibition improved hypogonadism in vivo.

**Figure 8:**
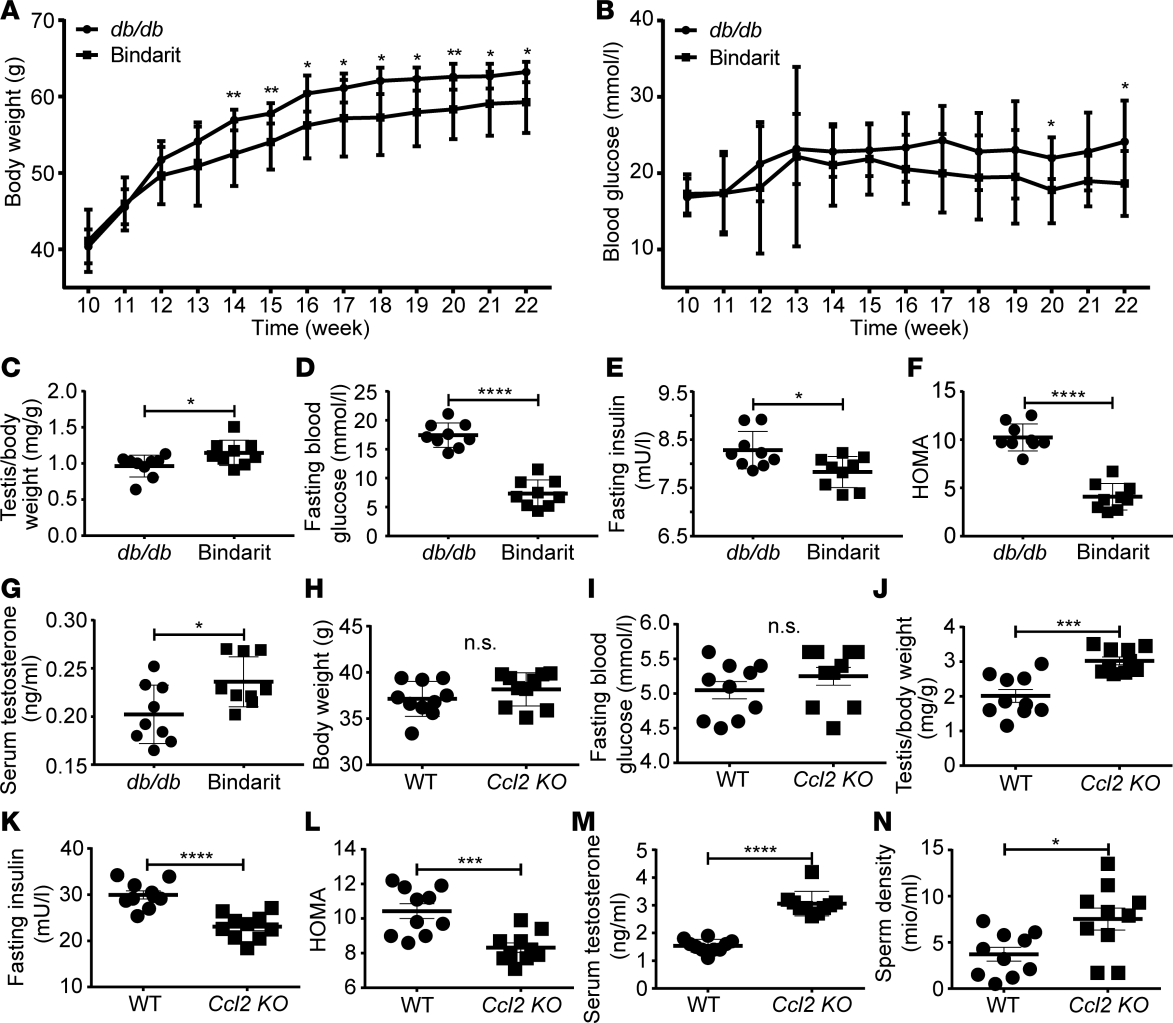
Pharmacological or genetic blockage of CCL2 ameliorated MetS and hypogonadism. (**A**–**G**) *db/db* mice were treated with vehicle or Bindarit (100 mg/kg/d) for 12 weeks. Weekly body weight (**A**), weekly random blood glucose levels (**B**), testis/body weight ratio at 12 weeks posttreatment (**C**), fasting blood glucose (**D**), fasting insulin (**E**), homeostatic model assessment (HOMA) index (**F**), and testosterone level in serum (**G**) were recorded and/or calculated 12 weeks post-treatment. (**H**–**N**) WT and *Ccl2-KO* mice were fed with a high-energy diet (HED) for 12 weeks. Body weight (**H**), fasting blood glucose (**I**), testis/body weight ratio (**J**) fasting insulin (**K**), HOMA index (**L**), serum testosterone (**M**), and sperm density (**N**) were recorded and/or calculated 12 weeks posttreatment. *N* = 9–14 mice in each group. Data are shown as means ± SEM. Student’s 2-tailed *t* test was used to compare means between 2 groups. *P < 0.05, **P < 0.01, ***P < 0.001, ****P < 0.0001.

Chemical inhibition and B6.129S4-*Ccl2*^tm1Rol^ (*Ccl2*-*KO*) mice were used to further support the role of CCL2 in MetS-related subfertility. First, we treated *db/db* mice with Bindarit (100 mg/kg/d) or vehicle control (0.5% methylcellulose) by oral gavage. The effects of Bindarit were most pronounced on obesity as it significantly decreased the body weight after 4 weeks of treatment (*P* < 0.05, Figure 8A). Significant decrease of blood glucose was observed in the treated group under random condition (*P* < 0.05, Figure 8B). The Bindarit-treated *db/db* mice showed higher testis/body weight ratio (*P* < 0.05, Figure 8C), thicker seminiferous epithelium (*P* < 0.0001), and more tubules with the highest Johnsen score (*P* < 0.001, Supplemental Figure 5, A–E), indicating less structural damage and a functional restoration in testes of the treated group (44). In addition, the treated *db/db* mice also had significantly reduced fasting blood glucose (*P* < 0.0001, Figure 8D) at the end of the experiment. Moreover, mice subjected to Bindarit had more favorable fasting serum insulin concentrations (*P* < 0.05, Figure 8E) as well as insulin sensitivity, with homeostatic model assessment (HOMA) index *P* < 0.001 (Figure 8F). Concomitantly, elevated serum testosterone levels suggested improved hypogonadism (*P* < 0.05, Figure 8G) in the Bindarit group as compared with that of controls. These results imply that CCL2 inhibition attenuated MetS symptoms, including partial rescue of hypogonadism in *db/db* mice. Next, we verified the findings using a high-energy diet (HED) model. *Ccl2*-*KO* mice were subjected to HED for 12 weeks. Age-matched WT mice were used as controls. Although there was no statistical difference in body weight (Figure 8H, P** = 0.23) or fasting glucose levels (Figure 8I, P** = 0.28), the *Ccl2*-KO group exhibited higher testis/body weight ratio (*P* < 0.01, Figure 8J), lower fasting insulin (*P* < 0.01, Figure 8K), and restored insulin sensitivity (HOMA index, *P* < 0.01, Figure 8L) as compared with HED-fed WT mice. Testosterone level (Figure 8M) and sperm density (Figure 8N) of the mutant mice were notably elevated. Consequently, *Ccl2-KO* group showed an improved IVF outcome as opposed to HED controls (Table 4). Together, these findings imply that genetic ablation of *Ccl2* was capable of ameliorating hypogonadism in HED-fed mice.

### CCL2 levels associated with hypogonadism.

**Figure 9:**
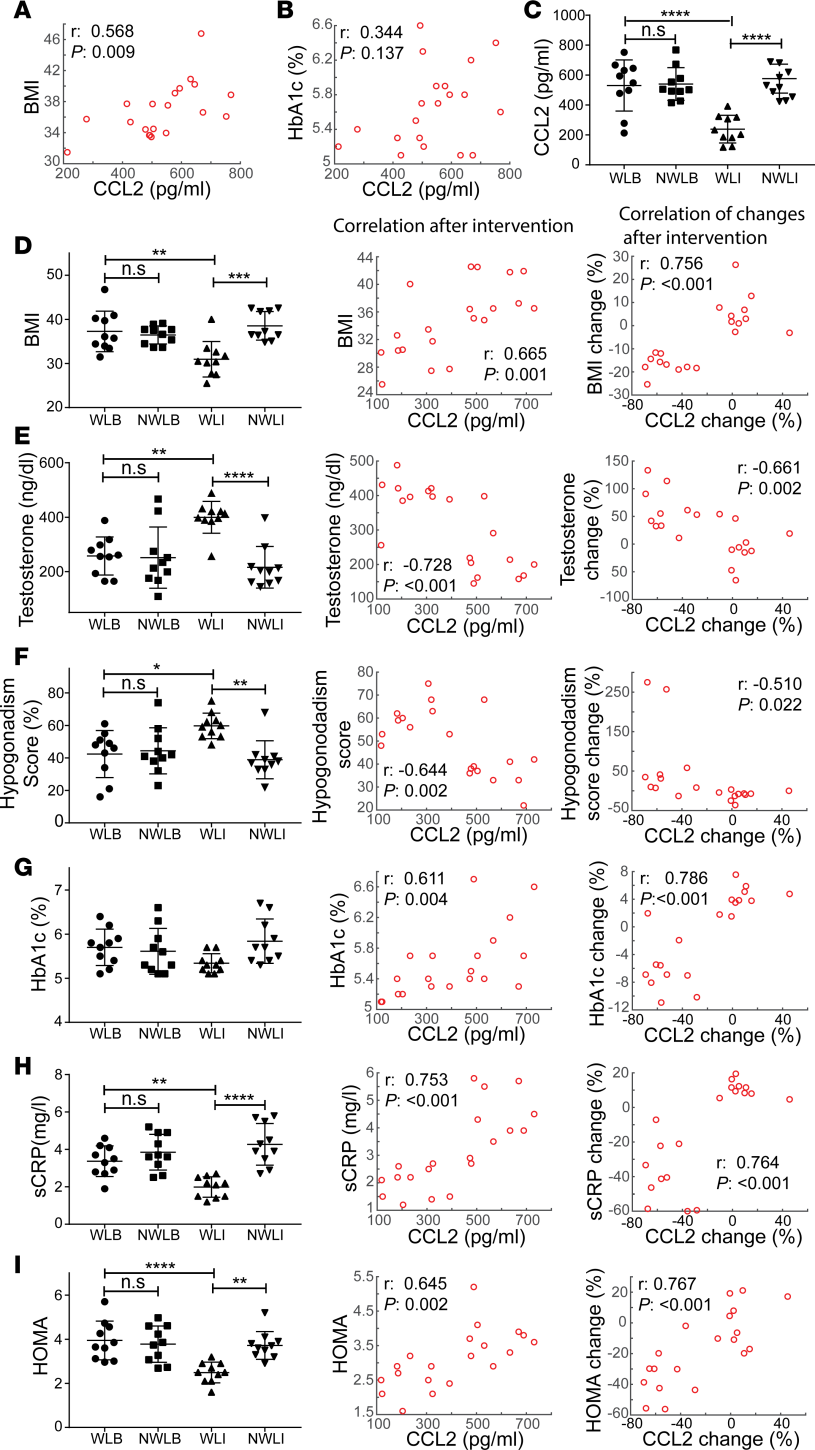
Clinical relevance of CCL2 in amelioration of MetS-related male hypogonadism. (**A** and **B**) Correlation of plasma CCL2 with BMI (**A**) and hemoglobin A1c (HbA1c) (**B**) at basal level in all participants (*n* = 20). (**C**) CCL2 levels in the weight loss after intervention group (WLI) was compared with its basal level (WLB), the non–weight loss after intervention group (NWLI) and its basal level (NWLB), respectively. (**D**–**I**) BMI (**D**), testosterone (**E**), hypogonadism score (**F**), HbA1c (**G**), sCRP (**H**), and HOMA (**I**) in different groups were compared at basal level and after intervention levels. Levels of each parameter after intervention were correlated to those of CCL2 (**D**–**I**, middle panels). Similar correlations were performed between changes of each parameter and those of CCL2 (basal vs. after intervention levels) (**D**–**I**, right panels). r, Pearson correlation coefficient. *N* = 10 patients in each group. Data are shown as means ± SEM. One-way ANOVA was used to compare means between 3 groups followed by Tukey’s post hoc comparisons. *P < 0.05, **P < 0.01, ***P < 0.001, ****P < 0.0001.

Infertile males participated in a weight reduction program as described in Supplemental Methods. At the start of the intervention, the mean body mass index (BMI) of all 20 participants (age 31.4 ± 5.3) was 36.9 ± 3.5 kg/m^2^, testosterone plasma levels were below normal range (255 ± 91, normal range 300–1200 ng/dL), and hypogonadism-related symptoms were observed throughout. The CCL2 level of this cohort was 535 ± 140 pg/mL (normal range 200–722 pg/mL) (Supplemental Table 3) and was positively correlated with BMI and hemoglobin A1c (HbA1c) (Figure 9, A and B). At the end of the weight reduction program, CCL2 level was significantly reduced in the weight loss after intervention group (WLI), compared with its basal level (weight loss basal, WLB) and the non–weight loss after intervention group (NWLI) (*P* < 0.0001) (Figure 9C). Moreover, after intervention, CCL2 levels were found to be not only highly and positively correlated with MetS components BMI, HOMA, and the proinflammatory factor sensitive C-reactive protein (sCRP) but also negatively correlated with hypogonadism indexes (testosterone and hypogonadism score). Similar correlations were observed between changes (basal vs. after intervention levels) of CCL2 and other parameters (Figure 9, D–I). These data suggest that in MetS, CCL2 is clinically relevant by predicting subjects’ recovery from symptoms of hypogonadism.

## Discussion

This study shows that chronic inflammation in full MetS can cause Leydig cell dysfunction and result in hypogonadism (Supplemental Figure 6). Increased IL-1β and CCL2 in adipose tissue and testes were associated with significant impairment of sperm quality and fertility as well as deterioration of androgen synthesis, which was caused by a clear reduction of Leydig cells. When exposed to IL-1β, Leydig cells showed enhanced CCL2 release, increased apoptotic signals, and dampened steroidogenesis. Inhibition of *Ccl2* transcription prevented Leydig cell dysfunction that was induced by inflammation in MetS in both in vitro and in vivo models. In a clinical cohort, the critical role of CCL2 in MetS-related hypogonadism was further supported by its significant correlations with testosterone and hypogonadism score. These findings indicate that therapeutic targeting of CCL2 expression might be effective in men with subfertility caused by MetS.

The impact of MetS on sperm function in males is controversial (45). Rodent models of MetS, including genetic, drug, and diet-induced models (38, 46–50), have been established and employed to address differences between rodents and humans. The *db/db* mouse model reflects the progress to full MetS characterized by concomitant type 2 diabetes mellitus along with obesity in a time-dependent fashion, and it is a validated and predictive animal model for the human condition (51). Indeed, we demonstrated in this study that *db/db* mice developed hypogonadism associated with MetS, which is closely comparable with the phenotypes in humans. In addition, mice made obese by feeding with HED showed reduced sperm motility and abnormal morphology, especially when elevated blood glucose levels occurred together with reduced blood testosterone concentrations (52). Hence, our findings in *db/db* mice were further supported by using the HED model.

It has been established that MetS is associated with inflammation (53) and that inflammation of the genitourinary tract results in suboptimal male fertility (54). However, whether chronic inflammation associated with MetS leads to reproductive dysfunction in males remained unclear. Previous studies showed that cytokines are present in both tubules and the interstitial compartment of the testis (31). When injected into adult rodent testis in vivo, these cytokines trigger effector mechanisms to directly inhibit STAR expression and steroidogenesis in Leydig cells (55). Administration of 100 ng IL-1β into the testes activates the JNK stress-related kinase pathway and increases E-selectin expression of microvascular endothelia and migration of immune cells (56). However, these investigations did not capture the main feature of MetS-related inflammation, the temporal and chronic increase of proinflammatory factors. Our results demonstrate that IL-1β not only is a potent inhibitor of Leydig cell function but also induces apoptosis of Leydig cells in culture. The source of IL-1β, however, is yet to be determined. As IL-1β is critically involved in the translation of obesity-related inflammation into diseases of adjacent organs (57–59), it is plausible to assume that the IL-1β derived from the adipose tissue contributes, at least in part, to the increased IL-1β level in the testes and to subsequent Leydig cell malfunction. Indeed, macrophages in the adipose tissue from obese mice showed elevated glycolysis and oxidative phosphorylation, which increased activation of HIF-1α and resulted in sustained local and systemic IL-1β production (60). Another potential source of IL-1β is the intratesticular cells, including macrophages and Leydig cells, that could be induced through either endocrine or paracrine signaling. Nevertheless, our results implicate the role of IL-1β in the crosstalk between adipose tissue and testis, which influences testicular structure and function in MetS.

Another insight from the present data is that the inflammation-mediated subfertility in MetS is achieved by induction of CCL2. CCL2 has been demonstrated to be a potential intervention point for the treatment of diabetes (61). In fact, we detected that it was increased in the testis of *db/db* mice as they progressed to obesity, adipose tissue inflammation, and infertility over a period of 24 weeks. Moreover, we observed that HED induced obesity in both WT and *Ccl2-KO* mice, but the latter showed higher testis/body weight ratio, testosterone level, and sperm density. These findings suggest that obesity-associated IL-1β carries out its detrimental effect by elevating CCL2. As one of the principal chemokines in the testes, CCL2 is primarily released from Leydig and peritubular cells (30, 31). Increased secretion of CCL2 would be expected to attract more monocytes, but this was not obvious with cell cytometry analysis in the testis of *db/db* mice in our study. This observation together with results of our in vitro models suggest that CCL2 impairs male fertility by inducing Leydig cell loss in a paracrine manner rather than by chemoattractantly recruiting monocytes. Indeed, injecting exogenous testosterone, which mimics Leydig cell function, can partially restore glucose intolerance and insulin resistance in male *db/db* mice (39). Previous findings showed that CCL2 directly induced podocyte apoptosis in diabetic conditions, which was significantly ameliorated by the inhibition of cysteine–cysteine chemokine receptor 2, a CCL2 receptor (62). The specific mechanism(s) of CCL2-induced apoptosis remains elusive. It has been shown that the cytotoxic effect of CCL2 is probably mediated by oxidative stress in retinal detachment–induced photoreceptor apoptosis (63). We observed several genes that were upregulated in the testis of *db/db* mice, including *Cdo1* (64, 65), *Fos* (66), and *Uchl1* (67), which have been reported to play roles in the oxidative stress response. Further, VDAC-1, a molecule that links apoptosis and oxidative stress (68), was regulated in human Leydig cells with CCL2. These results indicate that CCL2 likely exerts its cytotoxic effect through inducing oxidative stress.

As the numbers of both testicular macrophages and Leydig cells are reported to increase from birth to adulthood at a relatively constant rate, the current concept is that the two cell types are not only structurally but also functionally coupled under normal conditions (69). The reduction of F4/80^+^ macrophages might be caused by the reduction of Ins3L-positive Leydig cells in MetS. The lower percentage of M1 phenotype macrophages in the testis of *db/db* mice can be explained by the significantly increased levels of corticosterone in serum and TIF in *db/db* mice. Glucocorticoid release from the adrenal gland to the blood is facilitated in *db/db* mice with full MetS (70, 71), and glucocorticosteroids are widely known for their immunosuppressive role in chronic inflammatory diseases (72). In testes, corticosterone promotes polarization of macrophages toward the M2 phenotype and sustains immune privilege in the testes (73). Increased abundance of corticosterone in the testis of *db/db* mice explains the shift of the macrophage population to an antiinflammatory and more proapoptotic phenotype (73, 74) and fits the upregulated *Il-10* phenotype (75). This is a novel finding for the testis in subjects with MetS and will need further investigations. Nevertheless, upon chronic inflammation, the immune-privileged testis struggles to balance pro-and antiinflammation, in order to sustain normal functionality. Breaking this balance by inhibiting CCL2 expression could favor antiinflammation and improve reproduction in MetS-induced male subfertility.

Each MetS component can exert deleterious effects on male fertility separately. For instance, chronic inflammation in the adipose tissue of obese subjects correlates with the systemically increased production of reactive oxygen species (76, 77), which might diffuse into the testis and cause damages such as DNA fragmentation (78). Abnormal glucose homeostasis can interfere with the hypothalamo-pituitary-testicular axis and spermatogenesis and lead to reproductive impairment (79). Hyperglycemia contributes to endothelial dysfunction and results in organic erectile dysfunction (80), which might be partially responsible for the completely compromised fertility in *db/db* mice. Furthermore, dyslipidemia may induce poor sperm quality as increasing serum lipids are inversely associated with serum testosterone level and sperm parameters (81, 82). Although the pathogenesis of male subfertility in MetS is multifactorial and it is difficult to dissect the contribution from each one individually, it is plausible to assume that these components elicit combinatorial effects on male reproductivity. In this context, our present investigation indicates that promising outcomes can be achieved by targeting at least one of the complications of MetS.

The major limitation of our study is that we assumed the absence of Leydig cells after 12 weeks of age in the testis of *db/db* mice was caused by the chronic inflammation with development of MetS. This hypothesis was supported by our in vitro models, but Leydig cells exhibiting dysfunction and apoptosis were not seen in the testis of *db/db* mice in the present study. Future studies should use at least 1 more time point in between 6 and 12 weeks to determine the causes of Leydig cell loss in vivo. Similarly, macrophages also reduced in number in the testis of the *db/db* mice, which probably resulted from Leydig cell dysfunction and loss (83), and we were unable to further address the feedback loop of Leydig cells and testicular macrophages in the context of MetS because of the missing time points. Another limitation of this study is that the observations from *Ccl2*-*KO* mice might be caused by the constitutive whole-body deficiency of the *Ccl2* gene. A testis-specific *Ccl2*-*KO* mouse would allow us to further dissect the local effects of CCL2 from its systemic physiological impacts.

It has been well established that chronic inflammation plays a role in the pathogenesis of obesity-associated metabolic disease. While MetS potentially affects male reproduction, we have a limited understanding of how MetS-related inflammation exerts its detrimental effects on male fertility. In this study we demonstrate CCL2 as a preferentially MetS-induced molecule in testis with paracrine effects on Leydig cells. *db/db* mice display full MetS with obesity, adipose tissue inflammation, diabetes, and infertility. IL-1β and CCL2 suppress synthesis of androgens by inhibiting the activities of steroidogenic enzymes and ultimately induce apoptosis of Leydig cells. Altogether our study suggests that failed reproduction of *db/db* mice is likely a direct consequence of Leydig cell malfunction that is caused by increased CCL2 level and that inhibiting CCL2 expression may represent a therapeutic approach to ameliorate the reproductive dysfunction associated with MetS in males.

## Methods

### Animals and reagents.

Male BKS(D)-*Lepr*^db+/+^/JOrlRj (*db/db*) mice and B6.129S4-*Ccl2*^tm1Rol^ (*Ccl2-KO*) were obtained from Charles River Laboratories. The *db/db* mice with hyperglycemic (>11.1 mmol/L) fasting blood glucose levels were further examined. Age-matched BKS(D)-*Lepr*^db+/–^/JOrlRj (*db/+*) and C57BL6/N (WT) mice (Charles River Laboratories) served as controls. All animals were housed in groups of 3 to 4 animals at a temperature of 21°C ± 1°C with a 12-hour light/12-hour dark cycle with free access to food and water. In the Bindarit intervention experiment, *db/db* mice were treated daily by gavage from 10 to 22 weeks of age with vehicle (0.5% methyl-cellulose) or Bindarit (2-methyl-2-[[1-(phenylmethyl)-1H-indazol-3-yl]methoxy]-propanoic acid) (Cayman Chemical) at 100 mg/kg. The dosage of Bindarit was selected according to previous reports (84, 85). Body weight and random blood glucose were monitored weekly. Fasting (16 hours) blood glucose was recorded at the end of the experiment. Serum was collected for testosterone and insulin level analysis using testosterone chemiluminescence immunoassay kit (Beijing Furui Runkang Biotechnology Co. Ltd) and insulin chemiluminescence immunoassay kit (Beijing Furui Runkang Biotechnology Co. Ltd), respectively. Testes were isolated for histology study after sacrifice. In the HED experiment, *Ccl2-KO* mice, 6 weeks old, and age-matched WT mice were fed 5.228 kcal/kg, 16% calories from protein, 60% calories from saturated fat, and 24% calories from carbohydrates (Altromin) for 12 weeks. Body weight and fasting (16 hours) blood glucose were monitored weekly. At the end of the experimental period, animals were weighed at 8 am before euthanization by cervical dislocation under intraperitoneal ketamine/xylazine anesthesia. A total of 200–500 μL nonfasting blood was collected via the retroorbital venous plexus from all mice, and serum for biochemical determinations was generated by whole-blood centrifugation at 1300*g* for 10 minutes. Testis and EWAT were excised. Unless stated otherwise, all reagents were obtained from MilliporeSigma Chemie GmbH.

### Assessment of fertility.

Male *db/db* or WT mice were mated to female WT breeders in individual groups at 12 weeks of age. Formation of the vaginal plug was considered as a sign of successful mating. Females were then monitored for resulting pregnancies, and number of litters was recorded after birth.

### Assessment of mouse sperm parameters.

In order to isolate mouse sperm, cauda epididymis was excised and then rinsed with HS medium (135 mM NaCl, 5 mM KCl, 2 mM CaCl_2_, 1 mM MgCl_2_, 30 mM HEPES, 10 mM glucose, 10 mM lactic acid, and 1 mM pyruvic acid) and adjusted to pH 7.4 with NaOH. After transferring it to 1 mL of HS medium containing 5 mg/mL BSA and 15 mM NaHCO_3_, sperm were allowed to exude (15 minutes at 37°C, 5% CO_2_) from incisions (20–23). Sperm count was carried out using Neubauer Improved camber. Sperm motility (rapidly progressive, slowly progressive, nonprogressive, and immotile) was assessed according to human sperm parameter guidelines (20–23, 86).

### Superstimulation, cumulus-oocyte-complex collection, in vitro fertilization, and in vitro culture.

CD-1 female mice, 6 to 8 weeks old, from Charles River Laboratories, were injected with 5 IU equine chorion gonadotropin and with 5 IU hCG 42–46 hours later. Cumulus-oocyte-complexes were collected from the ampullae of females 13–15 hours after hCG injection. For each biological replicate, oocytes from 3 female mice were pooled, and sperm from males, *db/db*, *Ccl2*-*KO*, or WT, was used for IVF. Sperm was collected from the cauda epididymis. Gametes were coincubated in HTF medium (Merck) for 4 hours. Degenerated oocytes were counted and excluded from subsequent in vitro culture. Fertilized oocytes were washed and cultured under mineral oil in a 37°C humidified atmosphere employing modified KSOM medium (Merck). At day 4, cleavage and developmental rates up to the blastocyst stage were recorded. Experiments were repeated at least 6 times.

### Cell culture.

Mouse Leydig tumor cell line (MLTC-1) cells were provided by Eveline Baumgart-Vogt (University of Giessen, Giessen, Germany). Cells were maintained in RPMI medium (Gibco, Thermo Fisher Scientific) with supplementation of 10% fetal bovine serum and 1% Penicillin-Streptomycin-Glutamine (Thermo Fisher Scientific) (87). Primary HLC were cultured in Leydig Cell Medium (ScienCell). Cells were cultured at 37°C in 5% CO_2_ (*v/v*) in the presence or absence of stimulators, additives, and/or inhibitors: 1 IU hCG (Ferring), 1 ng/mL IL-1β (MilliporeSigma), and 100 μM Bindarit (Cayman Chemical). Cells were processed for total RNA, protein isolation, single-molecule fluorescence in situ hybridization (sm-FISH), or immunofluorescence as described below.

### Immunohistochemistry and immunofluorescence staining.

Whole testes from mice were fixed in Bouin’s fluid for 8 hours. Tissues were dehydrated in a graded series of ethanol and embedded in paraffin. Sections that were 7 μm thick were processed for stainings. Sections were deparaffinized and rehydrated before antigen retrieval, followed by processing for antibody staining against the Leydig cell marker protein INSL3 (orb18041, AA range: 10–50, 1:100, Biorbyt) or the macrophage marker protein F4/80 (MCA497GA, Cl:A3-1, 1:500, AbD). The stainings were developed using alkaline phosphatase (Dako, 1:200) followed by counterstain with Harris’ hematoxylin and mounting under VectaMount AQ (Vector Laboratories). For immunofluorescence on tissues, sections were immunostained with antibodies against STAR (8849, 1:1000, clone D10H12, Cell Signaling Technology) or CCL2 (orb36895, 1:1000, Biorbyt) antibodies followed by detection with secondary antibodies (anti–rabbit IgG, 711295152, 1:400, Jackson ImmunoResearch; or anti–mouse IgG, SAB3701033, 1:400, MilliporeSigma) and counterstained with DAPI. For immunofluorescence on cells, cell smears were fixed in 4% paraformaldehyde and permeabilized in 70% ethanol. After that, slides were immunostained with antibodies against STAR, CCL2, or VDAC-1 (SAB5201374, clone S152B-23, 1:200, MilliporeSigma) followed by conjugation with the abovementioned secondary antibodies and counterstain with DAPI.

### RT-qPCR.

Total RNA from tissue (stored in RNA*later* from Ambion, Thermo Fisher Scientific, at –80°C) or cell culture samples (harvested using RLT lysis buffer (QIAGEN) with 1% β-mercaptoethanol) was extracted using the RNeasy Mini Kit from QIAGEN according to the manufacturer’s instructions. RNA yield was quantified using a NanoDrop spectrophotometer (NanoDrop). Total RNA (1 μg) was used for reverse transcription in 20 μL reactions using the SuperScript III VILO kit (Invitrogen, Thermo Fisher Scientific). RT-PCR amplifications were performed using the IQ SYBR Green Supermix (Bio-Rad Laboratories GmbH) on the StepOne Plus real-time PCR system (Applied Biosystems, Thermo Fisher Scientific). Furthermore, RT2 Profiler PCR Array Mouse Male Infertility (QIAGEN) was applied to examine expression levels of fertility-related genes.

For PCR amplifications each well contained 5 μL SYBR Green, 3.2 μL RNase-free H_2_O, 0.3 μL primer, and 1.5 μL cDNA template. Cycling conditions were 95°C for 10 minutes, followed by 40 cycles at 95°C for 15 seconds, 60°C for 30 seconds, and 72°C for 30 seconds. The expression of each of the genes was measured in triplicate for each sample. PCR signal of the target transcript was normalized to the geometric mean of β-actin, and expression levels were assessed by relative quantification using the 2^-ΔΔCt^ method (88). After assessment of the 2^-ΔΔCt^ value, normalized data were corrected for outliers using the Grubb’s test. Data are expressed as fold changes in gene expression relative to WT control transcripts. A complete list of primers used can be found in Supplemental Figure 1.

### FISH.

sm-FISH probes were designed using an online tool available at https://www.biosearchtech.com/products/rna-fish 3′-amino modified oligonucleotides were obtained from LGC Biosearch. Pooled oligonucleotides were coupled to dyes and purified as described by Raj et al. (89). Fixed and permeabilized HLC were equilibrated in hybridization wash buffer (10% formamide in 2× SSC) and then incubated overnight in hybridization buffer (10% formamide, 10% dextran sulfate, 2 mM vanadyl-ribonucleoside complex, 0.02% RNase-free BSA, 0.001% *Escherichia coli* tRNA) with labeled mRNA probes at 37°C. After incubation, cells were washed twice in hybridization wash buffer for 10 minutes, followed by mounting and imaging. Analysis of mRNA molecules was carried out using a modified system developed by Raj et al. (89). A complete list of sm-FISH probe sets used can be found in Supplemental Figure 2.

### Western blot analysis.

Tissue was lysed using NP-40 lysis buffer containing 20 mM Tris/HCL (pH 7.5), 150 mM NaCl, and 1% (*v/v*) NP-40 plus protease and phosphatase inhibitor cocktail (Thermo Fisher Scientific), followed by incubation on ice for 20 minutes. Debris were removed by a 20,000*g* centrifugation at 4°C for 30 minutes. The supernatant was collected, and concentration of protein was determined with the Bradford Protein Assay (Bio-Rad). A total of 15 μg protein was separated by 8% SDS-PAGE and electrophoretically transferred from the gel to a polyvinylidene difluoride membrane (MilliporeSigma) by semidry blotting (Bio-Rad). The membrane was blocked in 1× TBS containing 0.1% Tween and 5% nonfat milk powder or BSA followed by overnight incubation with appropriate primary antibodies against STAR (8849, 1:1000, clone D10H12, Cell Signaling Technology), INSL3 (orb648755, 1:1000, Biorbyt), CCL2 (orb36895, 1:1000, Biorbyt), CYP17A1 (10443, 1:1000, clone E6Y3S, Cell Signaling Technology), and cleaved caspase-3 (9664, 1:1000, clone Asp175, Cell Signaling Technology), at 4°C. All membranes were washed 3 times with 1× TBS with 0.1% Tween-20 (TBST) and incubated for 30 minutes at room temperature with goat anti–rabbit IgG horseradish peroxidase–conjugated secondary antibody (1:3000; Dako code P0448). After another 3 washes in TBST, the proteins were detected with enhanced chemiluminescence system (Thermo Fisher Scientific) and visualized by FUSION-Solo (PEQLAB, VWR).

### Steroid concentrations in mouse.

TIF was collected from WT and *db/db* mice at 12–24 weeks of age. Testes were 50% decapsulated, placed into a 1.5 mL microcentrifuge tube with the exposed parenchyma up, and centrifuged (12,000*g*, 30 minutes, 4°C). Supernatants were collected, snap-frozen, and stored at –80°C until analysis. Collections averaged 4.0 ± 0.3 μL/100 mg testis. Gas chromatography–mass spectrometry (GC-MS) was performed to measure corticosterone and testosterone as described previously (90). Briefly, samples were equilibrated with deuterated internal standards, extracted using Extrelut NT columns, and purified using Sephadex LH-20 mini columns. Thereafter, heptafluorobutyrate derivatives were prepared. GC-MS was carried out on an Agilent Technologies 6890 series GC equipped with an Agilent Technologies 7683B automatic liquid sampler. The GC is directly interfaced to an Agilent Technologies 5975 inert XL mass selective detector. The following *m/z* ratios were measured for testosterone and corticosterone and their corresponding internal standards: *m/z* 680.4/683.4 for T/d3-T and 720.4/726.4 for B/d8-B.

### Flow cytometry (FACS) analysis.

For flow cytometry analysis testis were kept in ice-cold PBS after excision. Tunica albuginea was removed, and single-cell suspensions were retrieved after collagenase enzymatic digestion of seminiferous tubules. Briefly, tubules were dissociated in 1.5 mL of type A collagenase (Roche) and DNase (Roche) with 10% FCS (GIBCO) at 37°C for 30 minutes. Cell suspension was resuspended and filtered through a 70 μm cell strainer (BD). Red blood cells were removed by RBC lysis buffer (QIAGEN).

Testicular macrophage population was examined by FACSCanto II flow cytometer (Becton Dickinson). After exclusion of doublets, debris, and dead cells, immune cells were identified by using panleukocyte marker CD45 (103108, 2 μg/mL, clone 30-F11, BioLegend). Macrophages were identified using F4/80 (123116, 5 μg/mL, clone BM8, BioLegend) and CD64 antibodies (139304, 2 μg/mL, clone X54-5/7.1, BioLegend).

### Acridine orange flow cytometry.

Mouse epididymis was introduced to 800 μL of TNE buffer (150 mM NaCl, 10 mM Tris, 1 mM EDTA, pH 7.4) in a Petri dish at room temperature immediately after extraction. After that, the tissue was cut several times and spermatozoa were washed out. Two aliquots of 300 μL were flash-frozen in liquid nitrogen and stored at –80°C. The remaining sample was used for assessment of concentration and total sperm count.

Thawing of samples was done by diluting with the TNE buffer to adjust to 2 × 10^6^/mL and mixing thoroughly. The sample was placed on ice immediately after thawing. Measurement of all samples was carried out within 1 hour after thawing on FACSTrack flow cytometer (Becton Dickinson) as previously described by Evenson and Jost (91), with minor modifications to increase the sensitivity of the assay: 100 μL of sample kept on ice was mixed with 200 μL of acid detergent for 10 minutes and then stained by adding 600 μL of 6 μg/mL acridine orange in buffer and measured after 2.5 minutes. Samples were measured in batches of maximum 6 samples after equilibration of acridine orange within the flow cytometer and with adjusting of instrument settings according to the reference sample. All measurements were done in duplicates; a total of 5000 events excluding debris were collected in each run.

### ELISA.

IL-1β levels in whole testis lysates and CCL2 levels in mouse Leydig cell supernatant and whole testis lysates were measured using the mouse IL-1β ELISA kit (Wuhan Fine Biotech) or the mouse CCL2 ELISA kit (Wuhan Fine Biotech), respectively. In the clinical trial, the following indexes were determined: CCL2 (R&D Systems, Bio-Techne), testosterone (DRG Instruments), sCRP (Abcam), glucose (MilliporeSigma), insulin (Abcam), and HbA1c (Crystal Chem), following manufacturers’ instructions.

### Statistics.

Statistical analysis was performed using Graph Pad Prism 6 (GraphPad Software). Values in tables and graphs are expressed as mean ± SEM (unless otherwise specified). Data were examined for normality of distribution and variance homogeneity. Two groups of mice were compared with Student’s 2-tailed *t* test for independent data. Age-dependent mouse and cell culture parameters were analyzed with 1-way ANOVA to test the interaction between 3 or more groups. Where overall ANOVA showed significance, Tukey’s post hoc comparisons between treatments were performed. *P* < 0.05 (*P < 0.05, **P < 0.01, ***P < 0.001, ****P < 0.0001) was considered significant.

### Study approval.

Protocols for the Bindarit intervention experiments were approved by the Animal Research Committee of the Institute of Laboratory Animals, Chinese Academy of Medical Sciences Peking Union Medical College, Beijing, China. Protocols for the rest of the animal experiments were approved by the Animal Ethics Committee, University of Giessen. All animal experiments were conducted in accordance with the German Animal Welfare Act for the care and use of laboratory animals. Protocols for the clinical trial were approved by the Ethics Committee of Faculty of Medicine, University of Giessen.

## Author contributions

QJ, TL, and CW conceived and designed the experiments. QJ, CCM, CW, PH, SB, YC, MFH, SAW, APD, and HCS performed the experiments. QJ, CCM, TL, CW, MFH, SAW, PH, and LS analyzed the data. QJ, TL, CCM, SFP, and LS wrote and edited the manuscript. QJ and TL are the guarantors of this work and, as such, had full access to all the data in the study and take responsibility for the integrity and the accuracy of the data analysis.

## Supplementary Material

supplemental data

## Figures and Tables

**Table 4:**
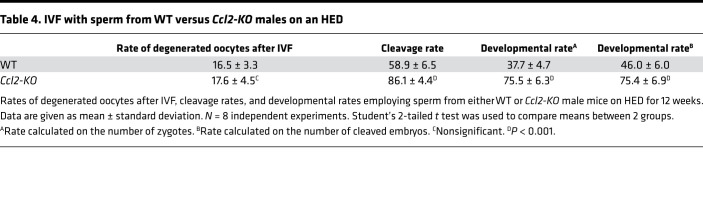
IVF with sperm from WT versus *Ccl2-KO* males on an HED

**Table 3:**
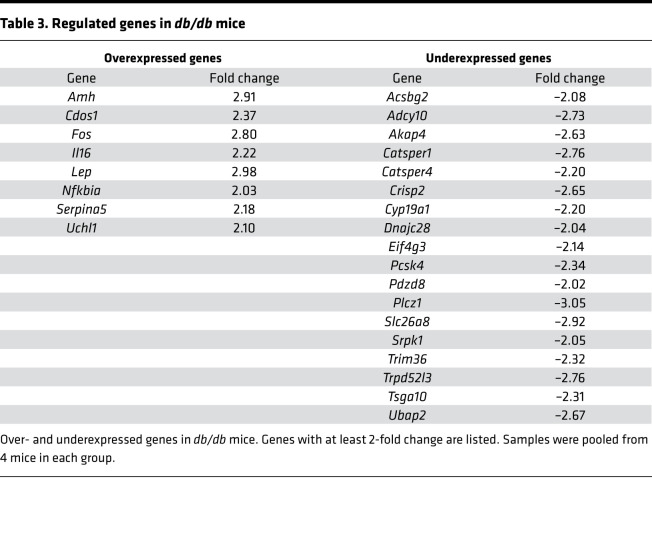
Regulated genes in *db/db* mice

**Table 2:**
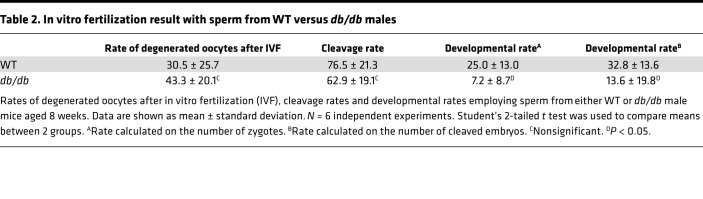
In vitro fertilization result with sperm from WT versus *db/db* males

**Table 1:**
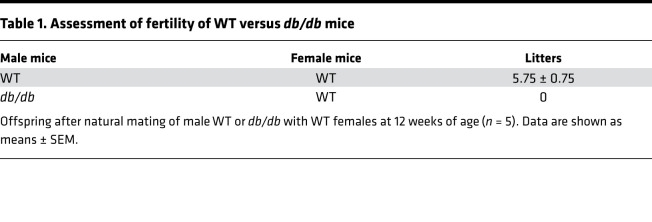
Assessment of fertility of WT versus *db/db* mice

## Version 1 11/05/2020

Electronic publication
