# Supplementary material for: Elevated CCL2 causes Leydig cell malfunction in metabolic syndrome
Source: JCI Insight. 2020 Nov 5;5(21):e134882. doi: 10.1172/jci.insight.134882 (PMC7710294; doi:10.1172/jci.insight.134882)
Supplement: supplemental data [file jciinsight-5-134882-s206.pdf]

Supplemental Data

Materials and Methods

Clinical trial in infertile males.

From couples admitting to fertility clinics male partners with a history of full metabolic syndrome (5 of 5 criteria fulfilled, increased waist hip ratio for central adiposity, hypertension, hypertriglyceridemia, decreased high-density lipoprotein (HDL) blood level, increased blood glucose level) were recommended to start a one-year program of weight reduction. They consented to blood retrieval and analyses as well as pseudonymized documentation of medical data and completing a questionnaire (1) for research purposes (NCT03977064, EC-No 205/16, EC of Faculty of Medicine).

In detail, total cholesterol, high-density lipoprotein (HDL) and triglyceride were measured by enzymatic assay (Fujifilm Wako Chemicals, Europe). Blood glucose levels were determined by the glucose oxidase method. Blood insulin was measured by immunometric assay. The estimate of insulin resistance by homeostasis model assessment (HOMA) score was calculated with the formula: fasting serum insulin (U/ml)*fasting plasma glucose (mmol/l)/22.5 (2). Body mass index kg/m^2 was computed from weight and height. Waist circumference between the rib cage and iliac crest with the subject in standing position was measured. Systolic and diastolic blood pressure were determined by mercury sphygmomanometer on the right arm after a five min rest. CCL2 serum levels were measured by enzyme-linked immunosorbent assay (R&D systems). Glycated hemoglobin was measured by a kit from Roche.

Calculations and statistics. From a total of 95 eligible participants in the program ten with more than 10% weight loss after 1 year were selected and compared with ten participants that had not achieved weight loss or even gained weight. 23 age-matched healthy males were taken as control. Comparisons of continuous and normally distributed variables were performed with analyses of variance for repeated measures.

Results

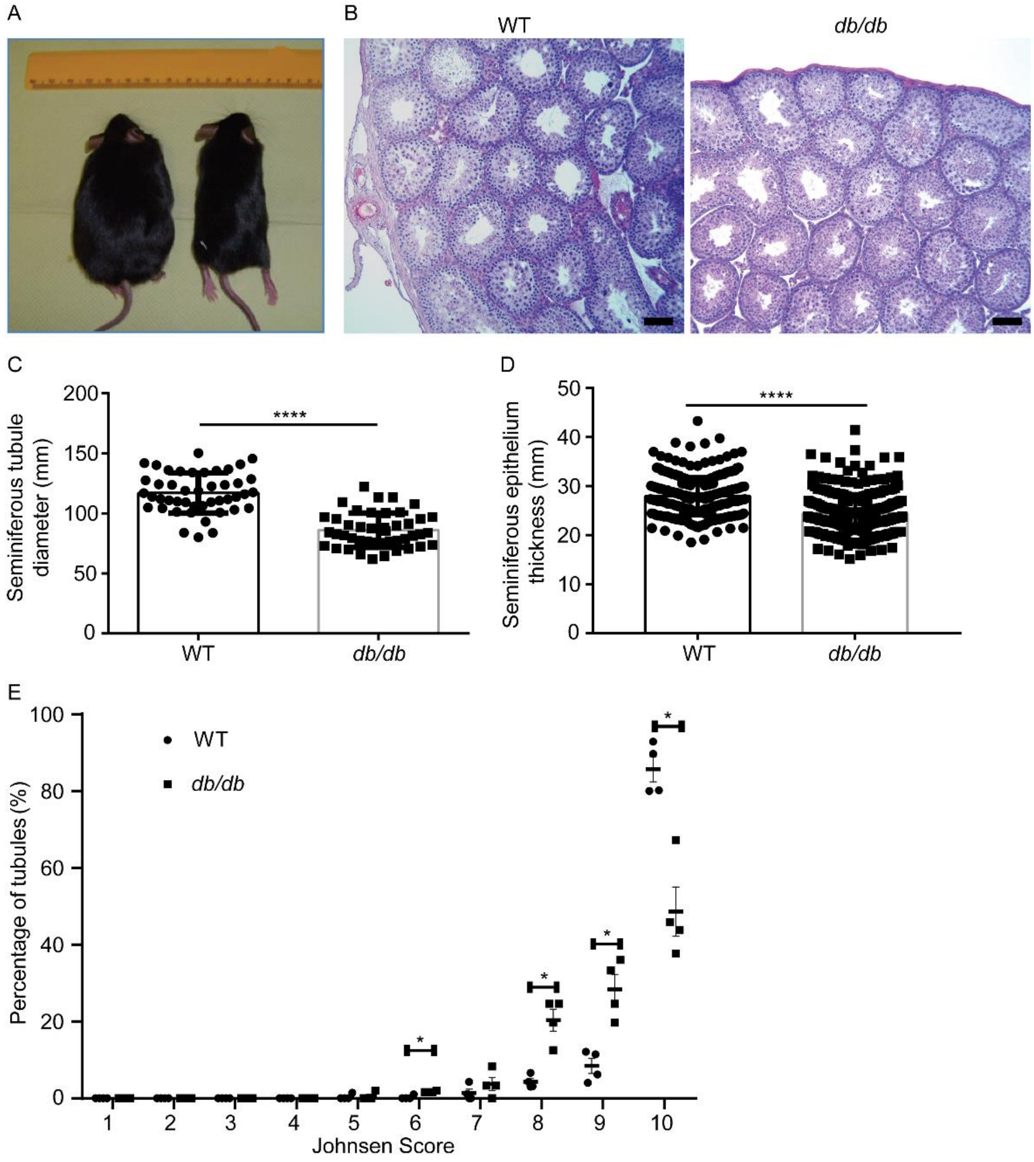

Supplemental Figure 1. Histological indexes of testicular architecture in WT and *db/db* mice.

(**A**) *db/db* (left) and wild type (WT) mice of 6 weeks. (**B**) Testicular sections stained by hematoxilin-eosin (H&E) from WT or *db/db* mice at 6 weeks. Scale bar: 100µm. (**C**) Randomly selected seminiferous tubules (n = 45 in each group) from WT and *db/db* mice of 12 - 24 weeks (n = 6 mice in each group) were subjected to measurement of seminiferous tubules' diameter. (**D**) Randomly selected seminiferous tubules (n = 289 in WT and n = 307 in *db/db* mice) from WT and *db/db* mice of 12 - 24 weeks (n = 6 mice in each group) were subjected to measurement of seminiferous epithelium thickness. (**E**) Randomly selected seminiferous tubules (n = 357 in WT and n = 239 in *db/db*) from WT and *db/db* mice of 12-

24 weeks (n = 4 mice in each group) were graded based on the Johnsen Score. Percentage of tubules with a specific Johnsen score were plotted to the y-axis for each group. Data represent mean ± SEM. Student's t test was used to compare means between two groups. *$P < 0.05$ and ****$P < 0.0001$.

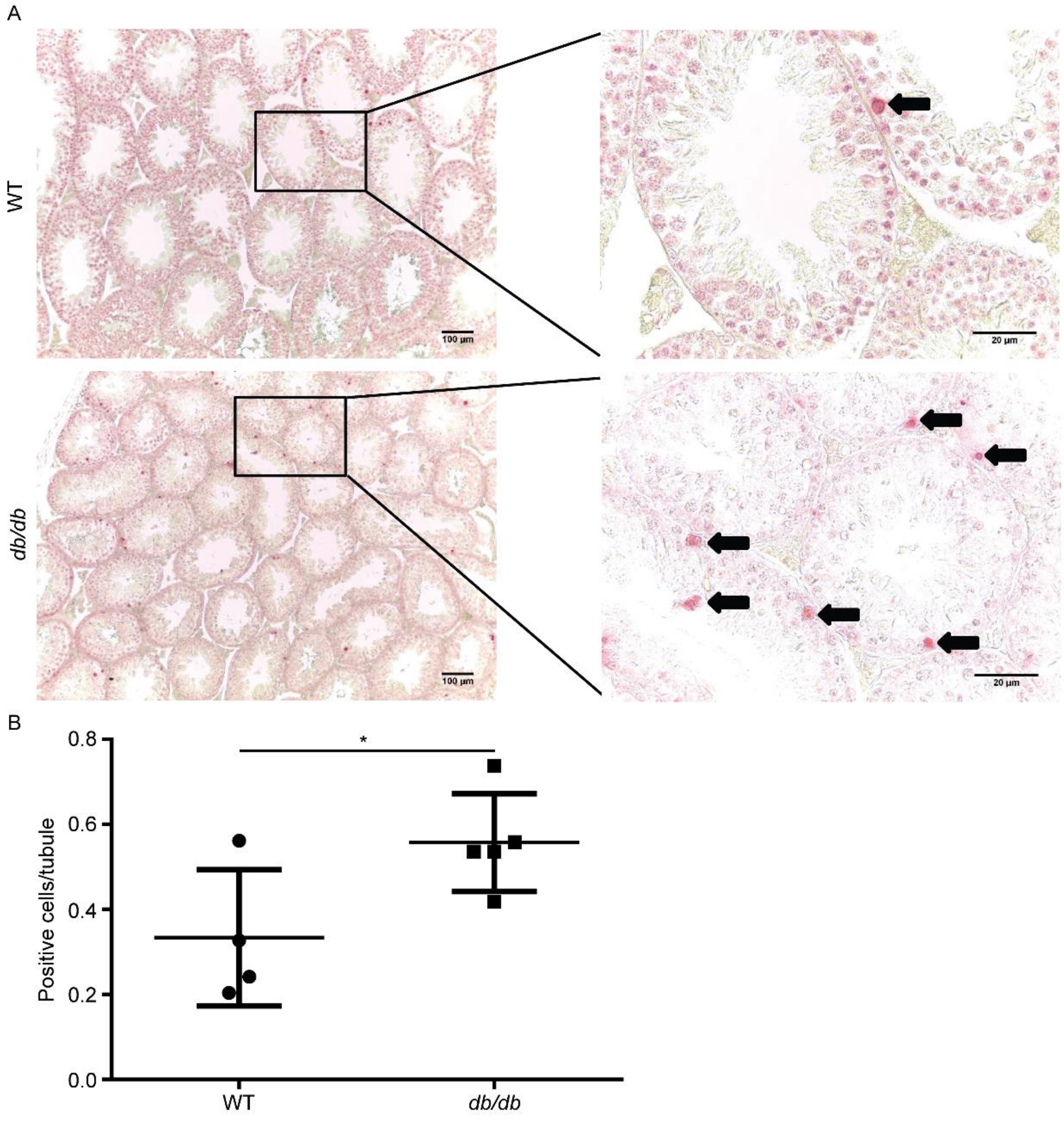

Supplemental Figure 2. *db/db* mice show increased testicular apoptosis.

(**A**) Apoptotic cells (arrows) in testes of WT (n = 4) and *db/db* (n = 5) mice at 12 - 24 weeks as determined by terminal deoxynucleotidyltransferase-mediated dUTP nick end labeling (TUNEL) assay. Positive cells per tubule were calculated and analyzed. Scale bar: 100µm (left) and 20 µm (right) (**B**) Data represent mean ± SEM. Student's *t* test was used to compare

means between two groups. *$P < 0.05$. The mean number of TUNEL-positive cells in testis of *db/db* mice was 0.56 per tubule, a 66.7% increase compared to the controls ($P < 0.05$).

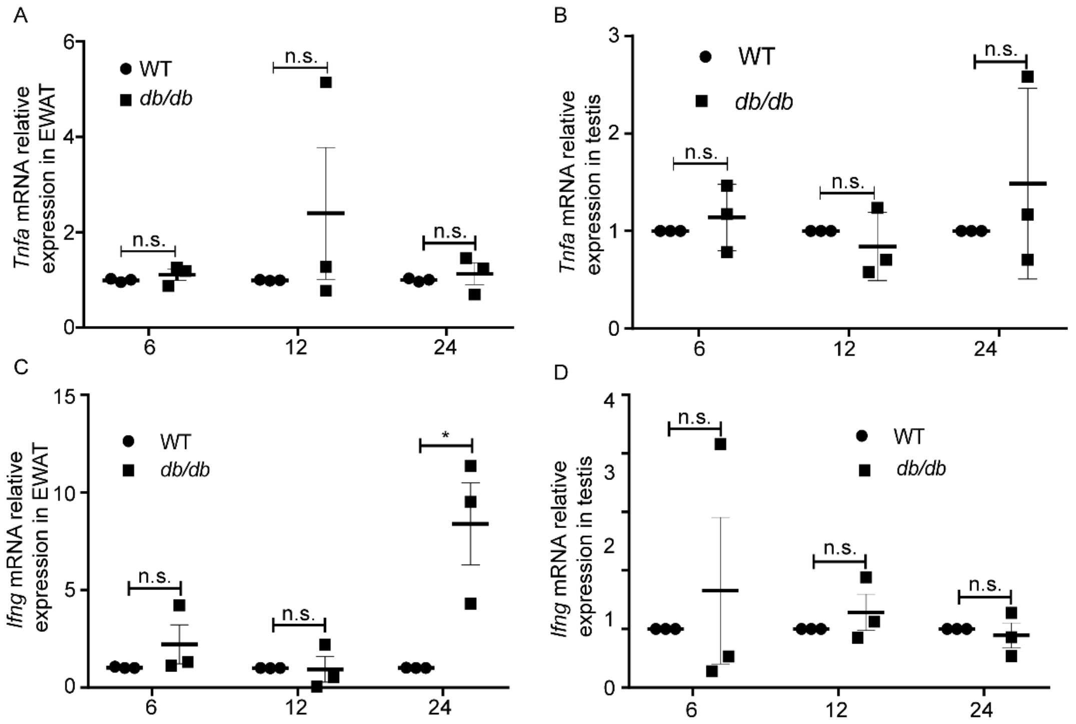

Supplemental Figure 3. Pro-inflammatory genes expression in testis.

(**A - D**) Quantitative real time PCR was performed to analyse the relative expression of *Tnfa* mRNA in epididymal white adipose tissue (EWAT) (**A**) and testes (**B**), *Ifng* in EWAT (**C**) and testes (**D**) of WT and *db/db* mice (n = 5). Data represents one of three independent experiments and are shown as means ± standard error of the mean (SEM). Student's *t* test was used to compare means between two groups. *$P < 0.05$, or nonsignificant (n.s.).

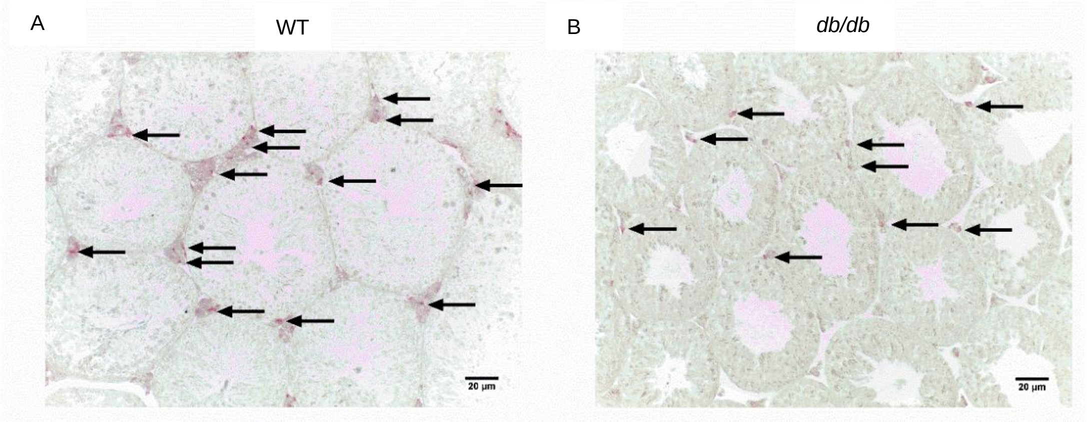

Supplemental Figure 4. Reduced macrophage marker expression in testis of diabetic *db/db* mice.

Representative immunohistochemical staining of macrophage surface protein F4/80 (arrows to interstitial tissue) from WT (n = 5) and *db/db* (n = 6) mice of 12 - 24 weeks is shown. (**A** and **B**) Significant decreased number of macrophages was found in the testis of *db/db* mice (**B**) compared to WT (**A**). Testis from four mice from each group were screened before selection of representative images. Scale bar: 20µm.

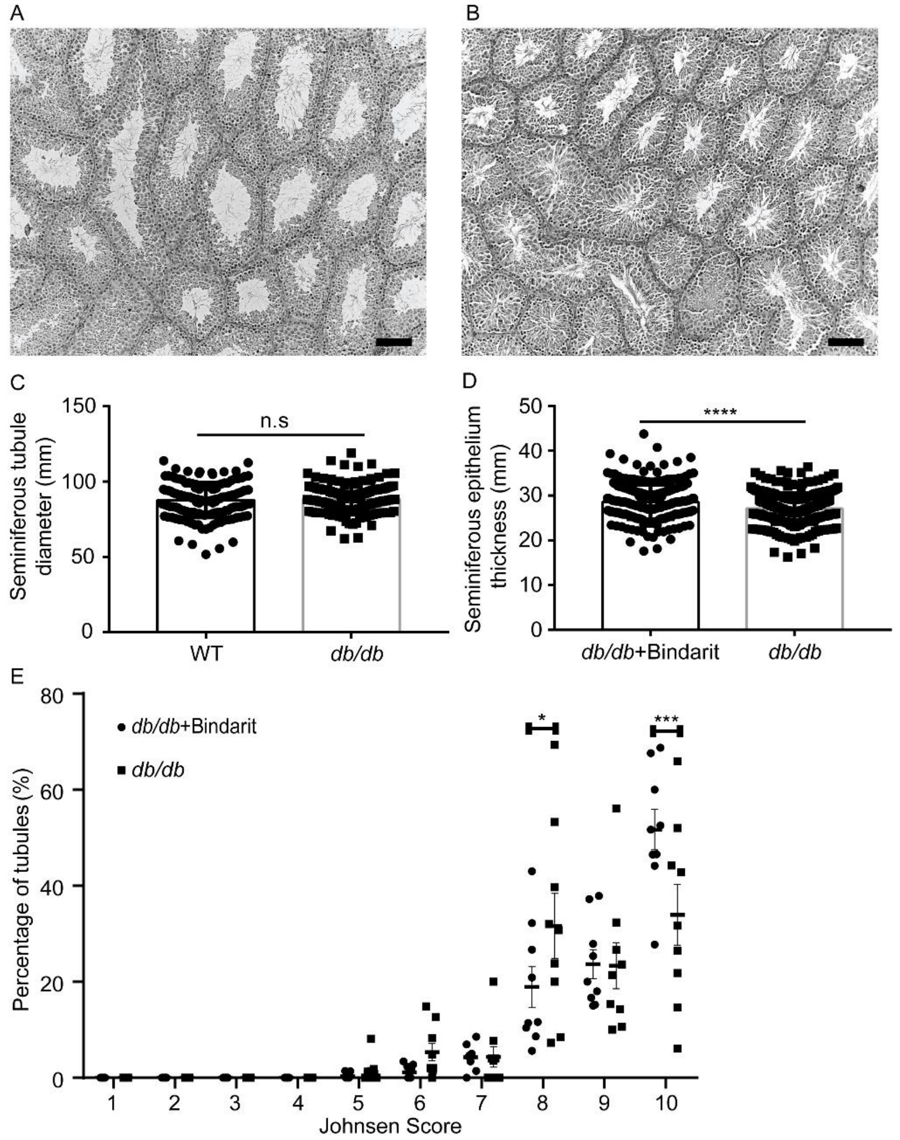

Supplemental Figure 5. Evaluation of histology and spermatogenesis index in *db/db* mice with or without Bindarit.

(**A** and **B**) Representative H&E stained sections from *db/db* mice without (**A**) or with (**B**) Bindarit treatment are shown. Scale bar: 100µm. (**C**) Randomly selected seminiferous tubules of *db/db* mice with (n = 153) or without Bindarit (n = 118) (n = 6 mice in each group) were subjected to measurement of seminiferous tubules' diameter. (**D**) Randomly selected seminiferous tubules of *db/db* mice with (n = 289) or without Bindarit (n = 284) (n = 9 mice in each group) were subjected to measurement of seminiferous epithelium thickness. (**E**) Randomly selected seminiferous tubules of *db/db* mice with (n = 489) or without Bindarit (n = 521) (n = 9 mice in each group) were graded based on the Johnsen Score. Percentage of tubules with a specific Johnsen score were plotted to the y-axis for each group. Mice were aged 12 - 24 weeks. Data are shown as mean ± SEM. Student's *t* test was used to compare means between two groups. *$P < 0.05$, ***$P < 0.001$, ****$P < 0.0001$, or nonsignificant (n.s.).

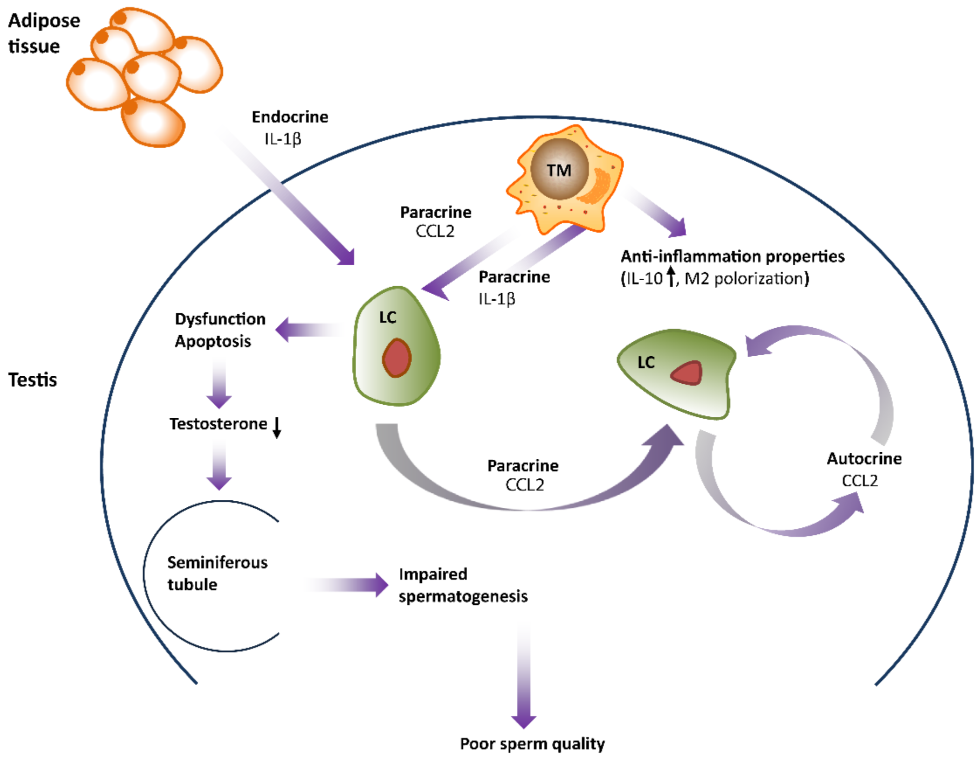

Supplemental Figure 6. Schematic representation of cellular interactions and factors in MetS-related male mice subfertility.

Chronic inflammation has been acknowledged as critical in the development of male subfertility. In the present study, MetS generated a chronic inflammatory condition in the testis, leading to damaged spermatogenesis and steroidogenesis, the latter was rescued by the *Ccl2* inhibition.

LC= Leydig cell, affected by CCL2 in paracrine or endocrine fashion.

TM = Testicular macrophage, can be a source of anti-inflammatory molecules, such as IL-10 and glucocorticoids.

Supplemental Table 1. Murine primer sequences for real time PCR

Genes		
Cyp11a1	Forward	5′-GTCGGAAGGTGTAGGTCAGG-3’
	Reverse	5′-CACTGGTGTGGAACATCTGG-3′
Cyp17a1	Forward	5′- GGGTATTGTGGATGTCCTGG-3′
	Reverse	5′- TCTGCATTCATCTTGGCTTG-3′
Il-1b	Forward	5′-AGGTCGCTCAGGGTCACAAG-3′
	Reverse	5′-GTGCTGCCTAATGTCCCCTTGAATC-3′
Il-10	Forward	5′-TAAGGCTGGCCACACTTGAG-3′
	Reverse	5′-GTTTTCAGGGATGAAGCGGC-3′
Ccl2	Forward	5′-CTGGATCGGAACCAAATGAG-3′
	Reverse	5′-CGGGTCAACTTCACATTCAA-3′
Star	Forward	5’-CAGGGAGAGGTGGCTATGCA-3’
	Reverse	5’-CCGTGTCTTTTCCAATCCTCTG-3’
Hsd17b1	Forward	5’-AAGCTCTTTCCTGCGATCAA-3′
	Reverse	5′- AGCTTCCAGTGGTCCTCTCA-3′

Supplemental Table 2. Human sm-FISH probes sequences

Probe#	Human *IL-1B* (5'-3')	Human *CCL2* (5'-3')
1	cttgtgcctcgaagaggt	tgtttctgggttagtctca
2	agagaatcccagagcagc	cgagcttcagtttgagaat
3	gctgcttcagacacttga	ctcgctggaggcgagagtg
4	ctcaggtacttctgccat	tgtttctgggttagtctca
5	agccatcatttcactggc	tgcgagcttcagtttgaga
6	caagtcatcctcattgcc	ttcatgctggaggcgagag
7	tagggccatcagcttcaa	acagaagggcggcagagac
8	cctggaaggagcacttca	tggctgctatgagcagcag
9	atccagagggcagaggtc	gagcccttggggaatgaag
10	gattcgtagctggatgcc	cattgattgcatctggctg
11	ttgctgtagtggtggtcg	taacagcaggtgactgggg
12	aacaactgacgcggcctg	cttcctattggtgaagtta
13	aggtctgtgggcagggaa	cgagcctctgcactgagat
14	cgttatcccatgtgtcga	ggtgattcttctatagctc
15	gcatcgtgcacataagcc	tctttgggacacttgctgc
16	gtgcagttcagtgatcgt	ggtcttgaagatcacagct
17	ttgctgtgagtcccggag	agatctccttggccacaat
18	ccagacatcaccaagctt	ttctgcttggggtcagcac
19	attcttttccttgaggcc	catggaatcctgaacccac
20	acacgcaggacaggtaca	tttgcttgtccaggtggtc
21	gctgtagagtgggcttat	caagtcttcggagtttggg
22	ttgggatctacactctcc	gggttgtggagtgagtgtt
23	gggcagactcaaattcca	aagttagctgcagattctt
24	atgtaccagttggggaac	ggggaaagctaggggaaaa
25	ttctgcttgagaggtgct	ggcataatgtttcacatca
26	ccaggaagacgggcatgt	aagcaatttccccaagtct
27	atatcctggccgcctttg	gaactgtggttcaagagga
28	ttgcatggtgaagtcagt	tcaaaacatcccaggggta
29	gctctctttaggaagaca	atgattcttgcaaagaccc
30	cctagggattgagtccac	tgggttgtggagtgagtgt
31	ctgttccctttctgccag	taagttagctgcagattct
32	gccgtactcaaaaacctt	ggggaaagctaggggaaaa
33	acaggaaagtccaggcta	ggcataatgtttcacatca
34	caggagatcctcttagca	aagcaatttccccaagtct
35	tgactgtcctggctgatg	gaactgtggttcaagagga
36	ggattggccctgaaagga	tcaaaacatcccaggggta
37	cctggctcaacaaaaggg	atgattcttgcaaagaccc
38	caggcgggctttaagtga	
39	ggagcgaatgacagaggg	
40	agcggttgctcatcagaa	
41	tacttcttgcccccctttg	
42	ggctcttttacagacact	
43	gagagcacaccagtccaa	

Index	BMI (kg/m^2)	CCL2 (pg/ml)	Testosterone (ng/dl)	HOMA	HbA1c (%)	sCRP (mg/L)	Hypogonadism Score (%)
MetS (fertile)	36.9± 3.5	535.5± 139.8	254.7±91.3	3.9±0.8	5.7±0.5	3.6±0.9	43±14
Control	23.8± 1.2^{****}	222.7± 28.2^{****}	725.3± 172.7^{****}	1.8± 0.5^{****}	4.6± 0.4^{****}	0.8± 0.3^{****}	$80.1±11.0^{****}$

Supplemental Table 3: Basal level of indexes of infertile males diagnosed with MetS and age-matched healthy controls in the clinical trial. N = 10. Comparisons were performed for each index by Student's *t* test. ****$P < 0.001$.

References

1. Alidjanov J, Wolf J, Schuppe HC, Weidner W, Diemer T, Linn T, et al. Validation of the German version of the 'Hypogonadism Related Symptom Scale' (HRS) in andrological patients with infertility, HIV infection and metabolic syndrome. *Andrologia.* 2014;46(10):1189-97.
2. Matthews DR, Hosker JP, Rudenski AS, Naylor BA, Treacher DF, and Turner RC. Homeostasis model assessment: insulin resistance and beta-cell function from fasting plasma glucose and insulin concentrations in man. *Diabetologia.* 1985;28(7):412-9.
